# Supplementary material for: Acetate Recapturing by Nuclear Acetyl-CoA Synthetase 2 Prevents Loss of Histone Acetylation during Oxygen and Serum Limitation
Source: Cell Rep. 2017 Jan 17;18(3):647–58. doi: 10.1016/j.celrep.2016.12.055 (PMC5276806; doi:10.1016/j.celrep.2016.12.055)
Supplement: Document S1. Supplemental Experimental Procedures, Figures S1–S6 [file mmc1.pdf]

Cell Reports, Volume *18*

Supplemental Information

Acetate Recapturing by Nuclear Acetyl-CoA Synthetase 2 Prevents Loss of Histone Acetylation during Oxygen and Serum Limitation

Vinay Bulusu, Sergey Tumanov, Evdokia Michalopoulou, Niels J. van den Broek, Gillian MacKay, Colin Nixon, Sandeep Dhayade, Zachary T. Schug, Johan Vande Voorde, Karen Blyth, Eyal Gottlieb, Alexei Vazquez, and Jurre J. Kamphorst

Experimental procedures

Estimation of acetate exchange fluxes

We denote by C, C_0, and C_2 the concentration of total acetate, ^{12}C acetate and U-^{13}C-acetate in the culture medium, respectively. We denote by c_0 the intracellular fraction of ^{12}C acetate and by V the packed cell volume. We denote by u, r, e=r-u the flux per cell volume of acetate uptake, release and exchange, respectively. We model the time dependent changes in the extracellular acetate concentration with the first order differential equations

(1) $$\frac{dC}{dt} = rV - uV$$

(2) $$\frac{dC_0}{dt} = rVc_0 - uV\frac{C_0}{C}$$

Integrating these equations over time, from the time that [$^{13}C_2$]-Acetate was added to the culture medium (t=0) to the final time (T) we obtain

(3) $$e = r - u$$

(4) $$xe = ar - bu$$

where

(5) $$e = \frac{C(T) - C(0)}{A}$$

(6) $$x = \frac{C_0(T) - C_0(0)}{C(T) - C(0)}$$

(7) $$A = \int_0^T dt V(t)$$

(8) $$a = \frac{1}{A}\int_0^T dt V(t) c_0(t)$$

(9) $$b = \frac{1}{A}\int_0^T dt V(t) C_0(t) / C(t)$$

Equation (5) is the definition of exchange rate. x in Eq. 6 quantifies the change in extracellular ^{12}C acetate relative to the change in total acetate. A (Eq. 7) is the area under the packed cell volume curve. a and b (Eqs. 8 and 9) are the average

intracellular and extracellular ^{12}C fractions, weighted by packed cell volume. Since the only source of ^{13}C acetate is the medium, the ^{13}C intracellular acetate fraction can be at most as large as the extracellular ^{13}C fraction and, therefore, the ^{12}C intracellular acetate fraction must be larger than the extracellular ^{12}C fraction:

(10) $$a > b$$

Solving the linear system of equations (3-4) for u and r we obtain

(11) $$u = e\frac{x-a}{a-b}$$

(12) $$r = e\frac{x-b}{a-b}$$

Since u and r are by definition nonnegative (larger or equal to zero), from equations 10-12, and the fact that a by definition must be smaller than 1, it follows that

(13) $$b < a < \min(1, x)$$

Parameter estimation

The parameters A, e, x and b can be estimated from experimental measurements of V, C and C_0 at different time points. The integrals (7) and (9) were estimated using the trapezium approximation. Given the experimental errors and lack of an experimental estimate of a, we sampled parameters using the following approach. We took V as measured and sampled C and C_0 from a Normal distribution with mean and standard deviation as measured. a was sampled uniformly from the range indicated by equation 13. Parameter sets (V,C,C_0,a) that did not satisfy (13) were rejected. Statistics was made over 1,000 sets of (V,C,C_0,a).

Estimation of fatty acid synthesis flux

The palmitate mass isotope distribution (MID) is modelled as follows. Palmitate MIDs are first corrected for natural abundance of ^{13}C. There is a M+0 pool of palmitate that was present in the cells at the time the tracer (or tracers) was added to the culture medium. The fraction of this pool at a given time t is denoted by ε_t. There is a pool of palmitate coming from *de novo* synthesis. This pool has a binomial MID distribution on n=8 acetyl groups each labelled at both carbons with probability p. The fraction of this pool at a given time t is denoted by $(1-\varepsilon_t)y$. Finally, there is a M+0 pool of palmitate coming from other sources. The fraction of this pool at a given time

t is denoted by $(1\text{-}\varepsilon_t)(1\text{-}y)$. Putting all together the palmitate MID distribution is given by

(Eq 1) $$x_i = \begin{cases} \varepsilon + (1-\varepsilon)\left[y(1-p)^n + 1 - y\right] & i = 0 \\ (1-\varepsilon)y\begin{pmatrix} n \\ m \end{pmatrix} p^m (1-p)^{n-m} & i = 2m \\ 0 & i = 2m+1 \end{cases}$$

The residual pool ε_t decays exponentially in time [X]

(Eq 2) $$\varepsilon_t = e^{-(k+\mu)t}$$

Where k is the palmitate turnover rate per unit of palmitate and μ is the cell population proliferation rate. From (Eq 1) and (Eq 2) we obtain

(Eq 3) $$x_i = \begin{cases} \alpha + (1-\alpha)(1-p)^n & i = 0 \\ (1-\alpha)\begin{pmatrix} n \\ m \end{pmatrix} p^m (1-p)^{n-m} & i = 2m \\ 0 & i = 2m+1 \end{cases}$$

where

(Eq 4) $$\alpha = 1 - (1 - e^{-(k+\mu)t})y$$

Given the palmitate MID (x_i) at a given time point, we calculate the mean squared error estimate of α and p. Given two time point estimates of α, we calculate the mean squared error estimate of $k+\mu$ and y. We fix one time point to an early time point where we already observe a saturation of p to a steady state value. The second time point run across measurements at later time points, obtaining time point estimates of $k+\mu$ and y. If these time points estimates do not change significantly in time we can warranty that with respect to the palmitate balance cells are in an approximate steady state. Finally, having estimated $k+\mu$, we estimate the palmitate synthesis rate as [X]

(Eq 5) $$f = C(k+\mu)$$

where C is the concentration of palmitate in cells.

ACSS1 overexpression

Human ACSS1 ORF sequence was obtained from NCBI database and was codon optimized and custom synthesized using GeneArt® Gene Synthesis (Life technologies). The sequence was cloned into pLIX mammalian expression vector obtained from Addgene (Addgene (https://www.addgene.org/41393/) and verified by DNA sequencing. Lentiviruses were produced by co-transfecting HEK293T cells with pLIX or pLIX+ACSS1 and the packaging plasmids. Supernatants containing virus were collected 24 and 48 hr after transfection, mixed with polybrene and used to infect MDA-MB-468 cells. Stable clones of MDA-MB-468 cells were selected for puromycin resistance and expression of ACSS1 was induced and maintained by the addition of 5μg ml^{-1} of doxycycline.

Total RNA extraction and quantitative RT-PCR

Total RNA was extracted using RNeasy kit (Qiagen) according to manufacturer's instructions. First strand cDNA was synthesized using 1 μg of total RNA using QuantiTect Reverse Transcription kit (Qiagen) according to manufacturer's instructions. Quantitative PCR (qPCR) was performed using SYBR® Green PCR Master Mix (BioRad) and primers for ACSS1 or for actin on a BioRad CFX96 real time PCR detection system (BioRad). Relative mRNA expression was calculated using the comparative Ct method after normalization to actin control.

SUPPLEMENTARY FIGURES

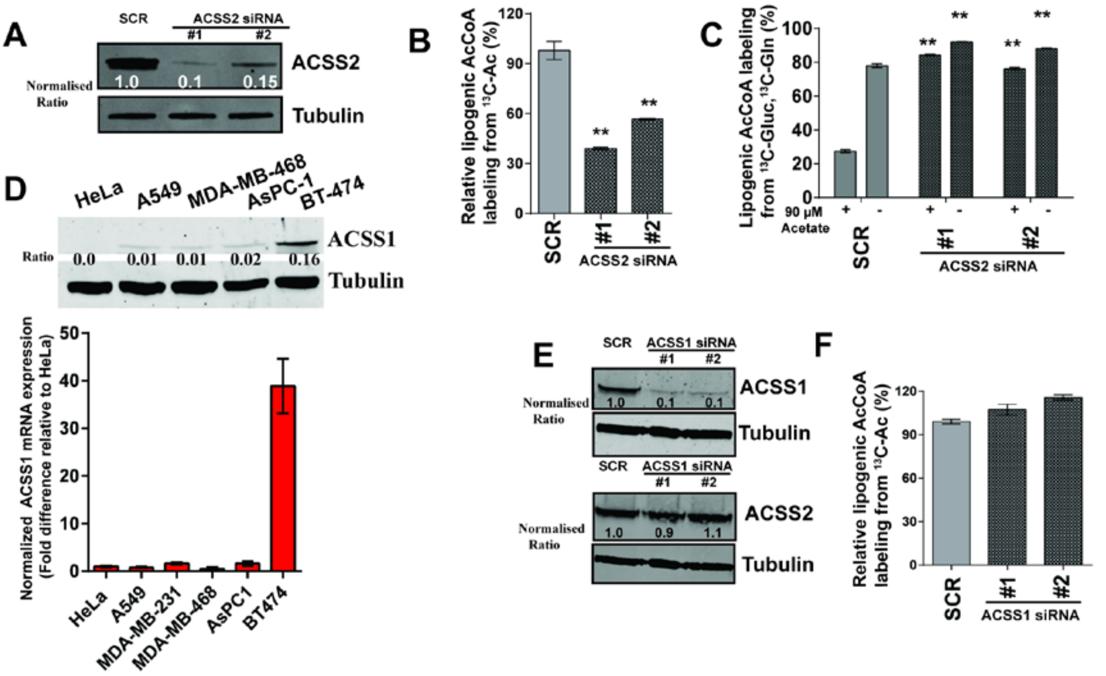

Figure S1. Related to Figure 1 (A) Western blot of ACSS2 in BT-474 cells transfected with either the scrambled RNA (SCR) or 2 independent ACSS2 siRNAs (#1 and #2). Expression levels of ACSS2 were normalized to tubulin and expressed relative to SCR control. (B) Percent ^{13}C labeling of lipogenic AcCoA from 90 µM U-^{13}C-acetate (Ac) in hypoxic BT-474 cells, transfected with either SCR or ACSS2 siRNAs (48 h labeling). (C) Percent ^{13}C labeling of lipogenic AcCoA from U-^{13}C-glucose and U-^{13}C-glutamine in hypoxic MDA-MB-468 cells with or without 90µM ^{12}C-acetate supplemented to the medium (48 h incubation). (D) Western blot analysis of ACSS1 expression in various cell lines, cultured under hypoxia (1% O_2) and low (1%) dialyzed serum for 48h. ACSS1 expression in each lane was normalized to tubulin and expressed as a ratio. Quantitative RT-PCR analysis of ACSS1 expression. Data was normalized to actin control and expressed relative to HeLa cells. (E) Western blot analysis of ACSS1 and ACSS2 expression in BT-474 cells, transfected with either the scrambled RNA (SCR) or 2 independent ACSS1 siRNAs (#1 and #2). Expression levels of ACSS1 or ACSS2 were normalized to tubulin in each lane and expressed as relative to SCR control. (F) Percent ^{13}C labeling of lipogenic AcCoA from 500µM U-^{13}C-acetate (Ac) in hypoxic BT-474 cells transfected with SCR or independent ACSS1 siRNAs (48 h labeling). For B, C, D and F, data are means ± SD (n=3). ** $p < 0.01$.

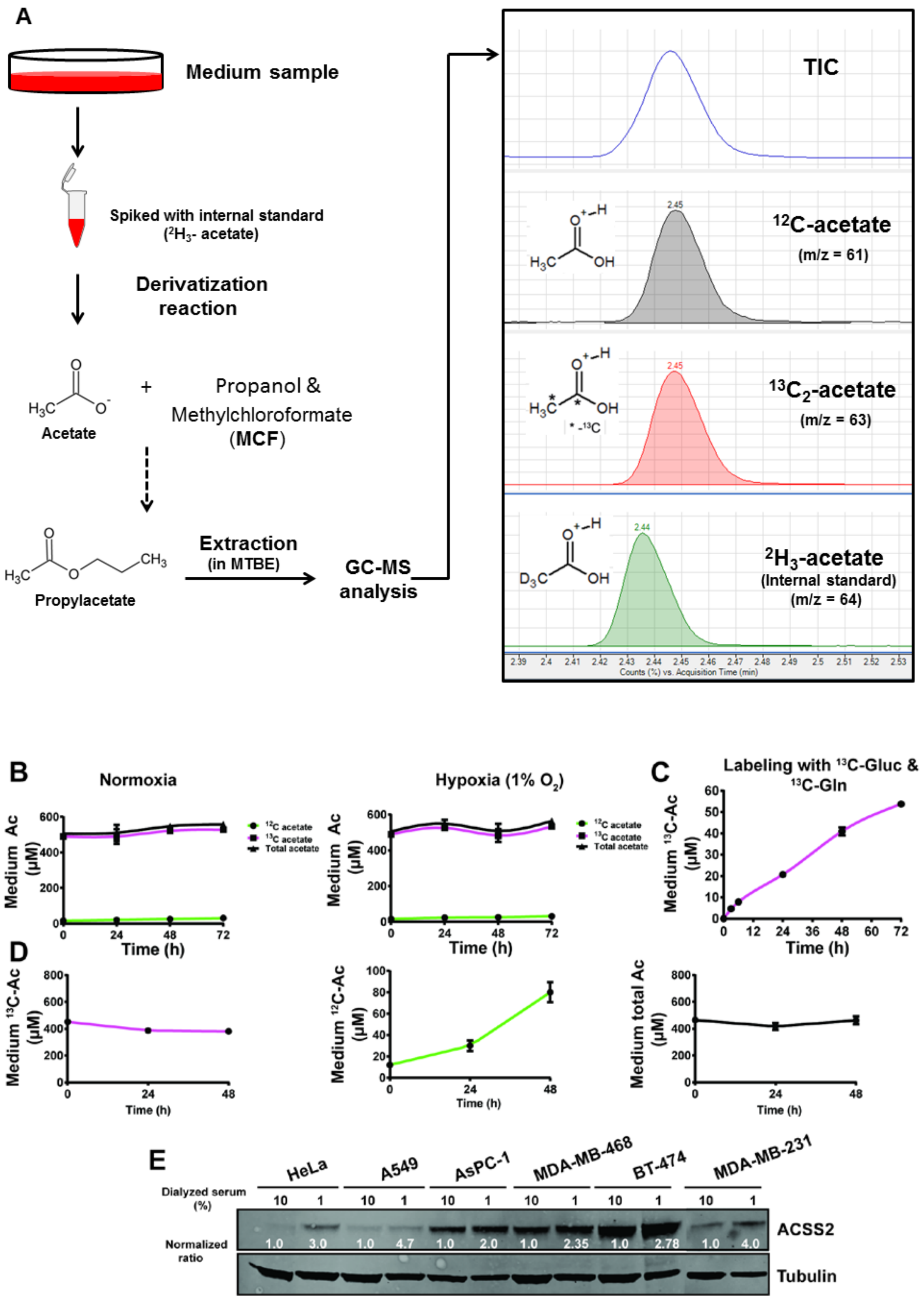

Figure S2. Related to Figure 2. (A) Scheme of acetate quantification from media samples by GC/MS. Culture medium was sampled and spiked with $^2H_3$-acetate as an internal standard and then derivatized to propyl-acetate in a methyl chloroformate

(MCF)-driven reaction (details in methods). Following extraction in MTBE (Ter-butyl methyl ether), samples were analyzed by GC/MS. Shown are the total ion current (TIC) and the integrated extracted ion chromatograms for ^{12}C-acetate (m/z 61), $^{13}C_2$-acetate (m/z 63) and $^{2}H_3$-acetate (m/z 64), which are generated from propyl-acetate by the electron impact ionization. Numbers on top of the peaks represent retention times. (B) Time course of U-^{13}C-acetate, ^{12}C-acetate and total acetate concentrations in experiment medium (10% dialyzed serum) incubated without cells. (C) Time course of ^{13}C acetate concentration in the medium of MDA-MB-468 cells incubated with U-^{13}C-glucose and U-^{13}C-glutamine. (D) Time course of U-^{13}C-acetate, ^{12}C-acetate and total acetate concentrations for hypoxic MDA-MB-468 cells cultured in medium containing physiological glucose (5.5 mM) and glutamine (0.65 mM), and additionally 10% dialyzed serum and 500 µM U-^{13}C-Acetate. (E) ACSS2 expression in panel of cancer cell lines, under hypoxia (48 h incubation) and high (10%) or low (1%) dialyzed serum. Band intensities of ACSS2 in each lane were first normalized to tubulin and then expressed as ratio normalized to 10% dialyzed serum condition for each cell line to see the fold increase in 1% dialyzed serum condition. For B-D, data are means ± SD (n=3).

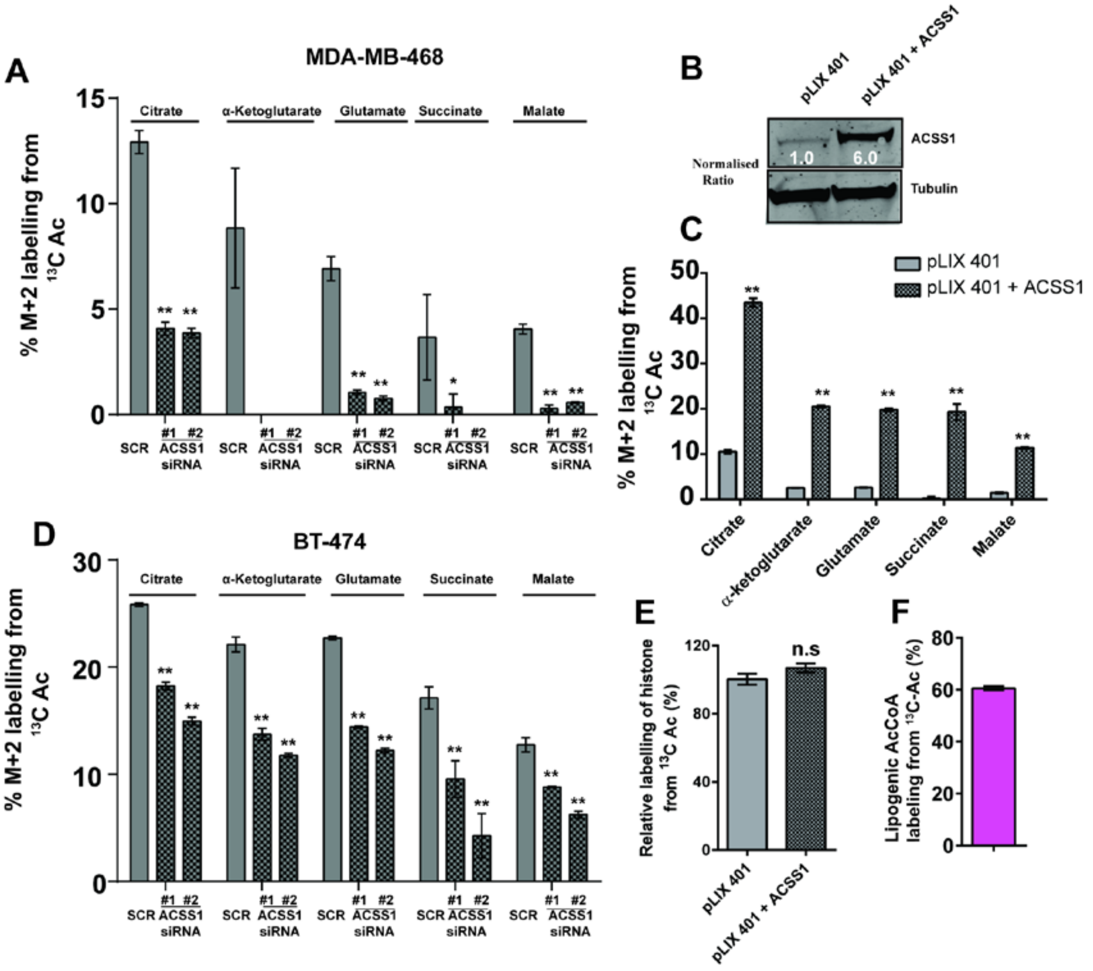

Figure S3. Related to Figure 3. ACSS1 knockdown by two independent siRNAs (#1,#2) decreases labelling of TCA cycle intermediates in MDA-MB-468 (A) and BT-474 (D) cells from 500µM U-^{13}C-acetate. (B) Western blot of ACSS1 in MDA-MB-468 cells stably transfected with either empty vector (pLIX 401) or vector with ACSS1 ORF (pLIX 401 + ACSS1). Cells were maintained in 5µg ml^{-1} doxycycline for induction of ACSS1 expression. (C) Labelling of TCA cycle intermediates for 48h from 500µM U-^{13}C-acetate in MDA-MB-468 cells stably transfected with pLIX 401 or pLIX 401+ ACSS1. Cells were maintained in 5µg ml^{-1} doxycycline for induction of ACSS1 expression. (E) Relative labelling of histone bound acetate from 500µM U-^{13}C-acetate in MDA-MB-468 cells stably transfected with pLIX 401 or pLIX 401+ ACSS1. Cells were maintained in 5µg ml^{-1} doxycycline for induction of ACSS1 expression. (F) Percent ^{13}C labeling of lipogenic AcCoA from 500µM U-^{13}C-acetate (Ac) in MDA-MB-468 cells grown under hypoxia and low serum conditions (48 h labeling). All data are means ± SD (n=3), * $p < 0.05$, ** $p < 0.01$.

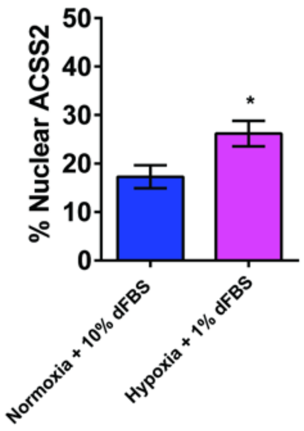

Figure S4. Related to Figure 4. Quantification of the nuclear fraction of ACSS2 (%) in BT-474 cells cultured under normoxia and 10% serum or hypoxia and 1% serum conditions for 48h. Data are means and SD of 3 independent experiments (8 images per experiment). * $p < 0.05$.

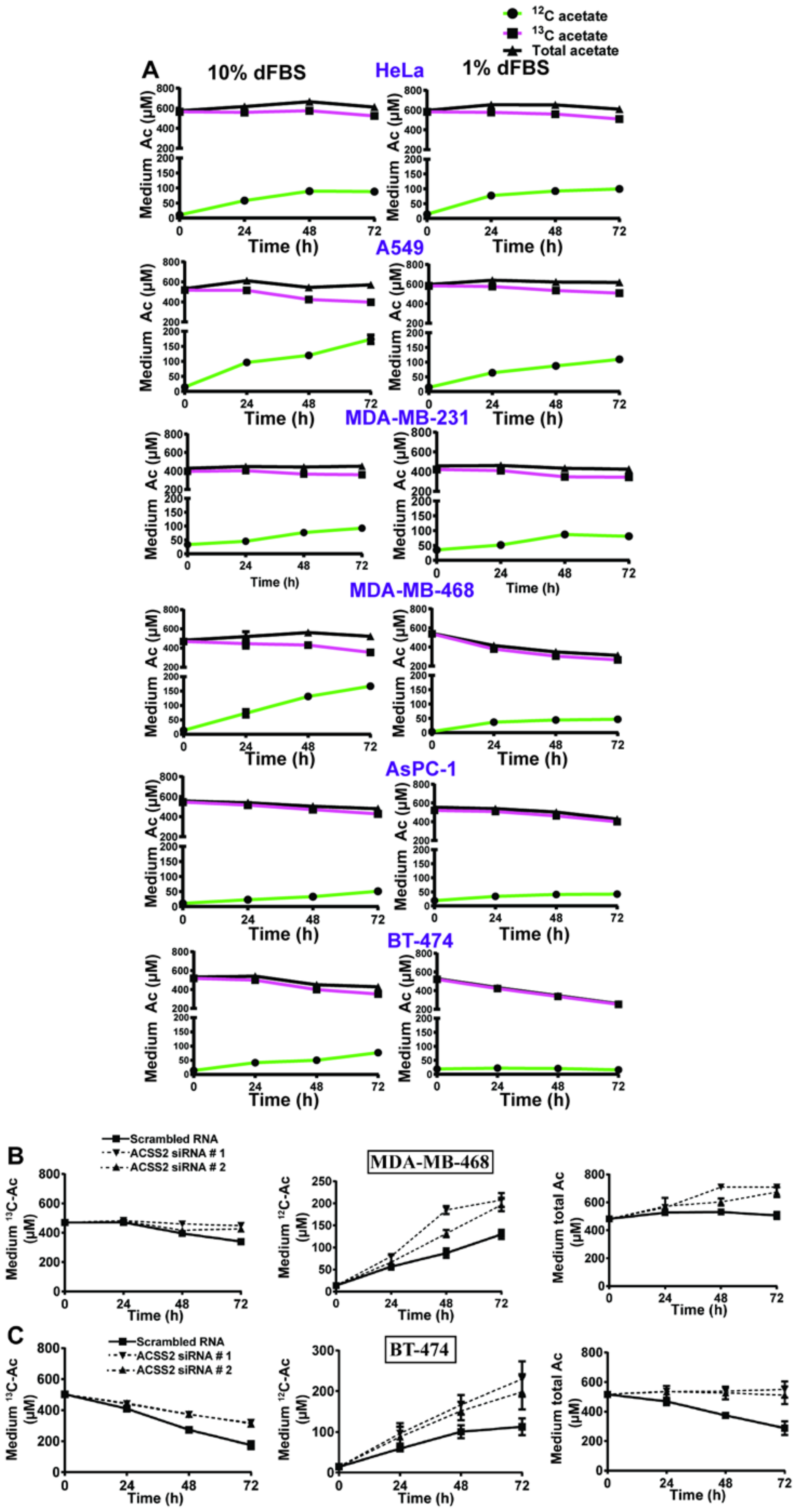

A
10% dFBS
1% dFBS
12C acetate
13C acetate
Total acetate
HeLa
A549
MDA-MB-231
MDA-MB-468
AsPC-1
BT-474
Medium Ac (μM)
Time (h)
B
MDA-MB-468
Scrambled RNA
ACSS2 siRNA # 1
ACSS2 siRNA # 2
Medium 13C-Ac (μM)
Medium 12C-Ac (μM)
Medium total Ac (μM)
C
BT-474

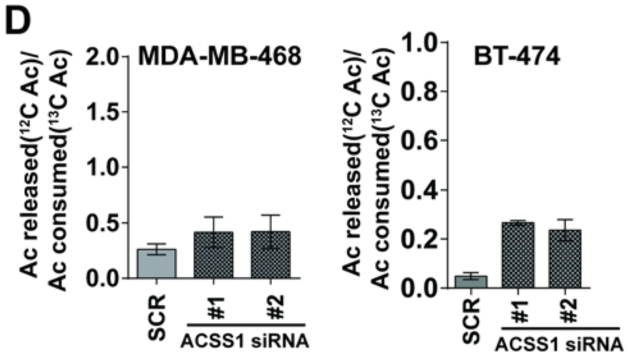

Figure S5. Related to Figure 5. (A) Time course of U-^{13}C-acetate, ^{12}C-acetate and total acetate concentrations for panel of cancer cell lines incubated under hypoxic conditions in medium supplemented with 10% or 1% dialyzed serum. (B) Acetate exchange by MDA-MB-468 cells following SCR or ACSS2 siRNAs under hypoxic conditions in medium supplemented with 10% dialyzed serum and 500μM U-^{13}C-acetate. (C) Same but for BT-474 cells. (D) Acetate exchange by MDA-MB-468 and BT-474 cells following SCR or ACSS1 siRNAs under hypoxic conditions in medium supplemented with 1% dialyzed serum and 500μM U-^{13}C-acetate. All data are means ± SD (n=3).

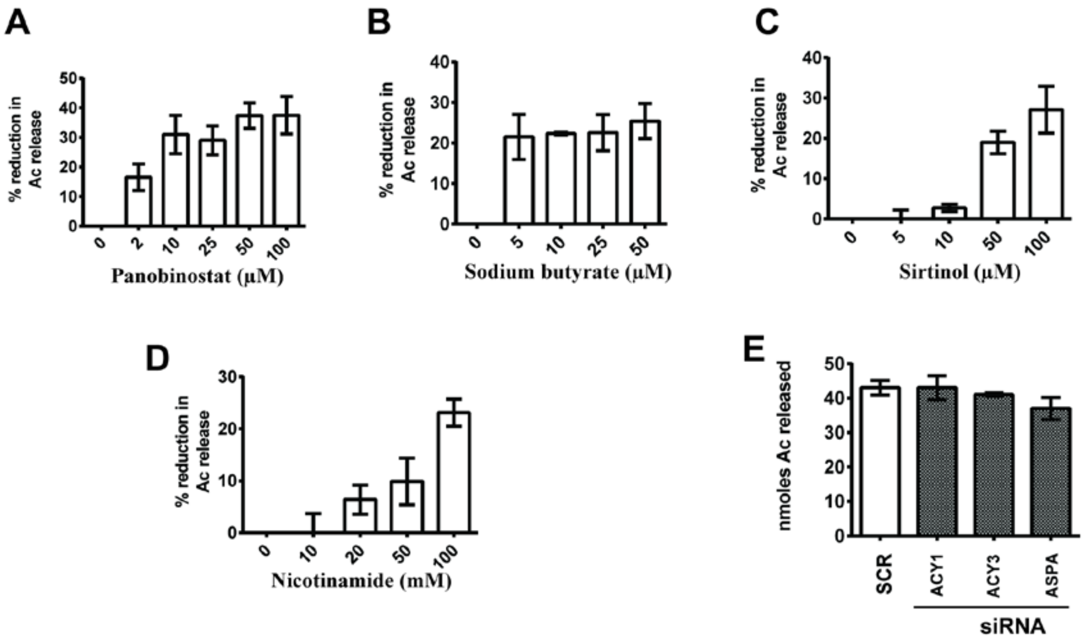

Figure S6. Related to Figure 6. (A) Percent reduction in acetate released by MDA-MB-468 cells treated with increasing concentrations of panobinostat. Cells were incubated with indicated concentrations of panobinostat for 6h in hypoxia in medium containing 1% dialyzed serum and 500µM U-^{13}C-acetate. Data is expressed as % reduction in ^{12}C acetate release relative to controls. (B) Same as (A), but with sodium butyrate treatment at indicated concentration (C) Same as (A), but with sirtinol treatment at indicated concentrations. (D) Same as (A), but with nicotinamide treatment at indicated concentrations. (E) Plot of percent reduction in acetate released by MDA-MB-468 cells treated with different siRNAs of amino acid deacetylases. Cells were transfected with either the scrambled siRNA or different SMARTpool siRNAs targeting aminoacylase 1 (ACY1), aminoacylase 3 (ACY3) or aspartoacylase (ASPA). Cells were incubated for 6h in experiment DMEM with 1% dialyzed serum. For A-E, data are means ± SD (n=3).
