# Supplementary material for: Acetate Recapturing by Nuclear Acetyl-CoA Synthetase 2 Prevents Loss of Histone Acetylation during Oxygen and Serum Limitation
Source: Cell Rep. 2017 Jan 17;18(3):647–58. doi: 10.1016/j.celrep.2016.12.055 (PMC5276806; doi:10.1016/j.celrep.2016.12.055)
Supplement: Document S2. Article plus Supplemental Information [file mmc2.pdf]

Cell Reports

Article

Acetate Recapturing by Nuclear Acetyl-CoA Synthetase 2 Prevents Loss of Histone Acetylation during Oxygen and Serum Limitation

Graphical Abstract

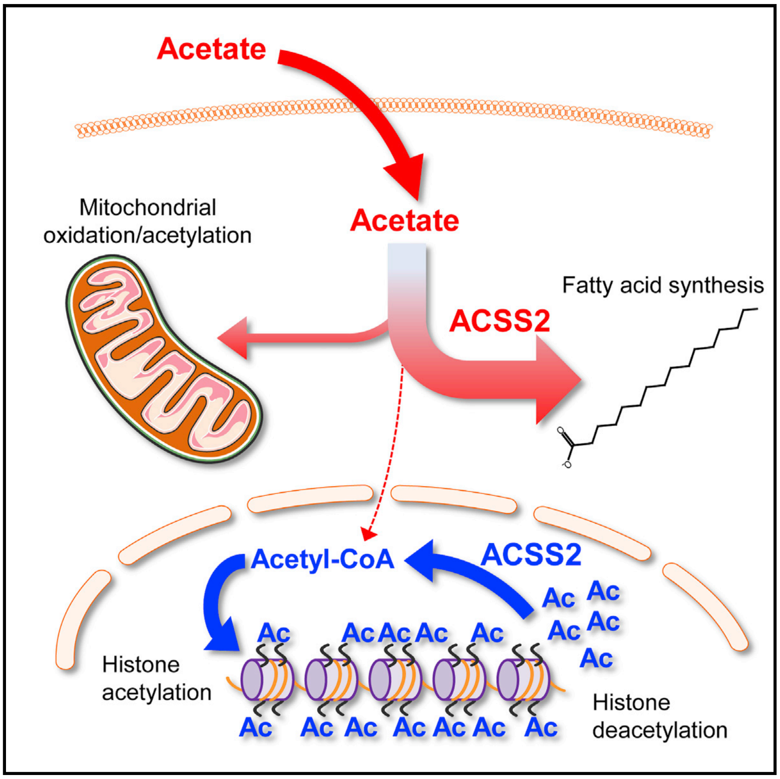

Highlights

- Acetyl-CoA synthetase 2 (ACSS2) expression controls acetate uptake and utilization
- Exogenous acetate is used for fatty acid synthesis rather than histone acetylation
- The main function of nuclear ACSS2 is to retain acetate released from histones
- Nuclear and cytosolic acetyl-CoA pools are largely compartmentalized

Authors

Vinay Bulusu, Sergey Tumanov, Evdokia Michalopoulou, ..., Eyal Gottlieb, Alexei Vazquez, Jurre J. Kamphorst

Correspondence

jurre.kamphorst@glasgow.ac.uk

In Brief

Acetyl-CoA synthetase 2 (ACSS2) generates acetyl-CoA from acetate and is important for tumor growth. Bulusu et al. show that, in hypoxic tumor regions, ACSS2 is prominently expressed in the nuclei of tumor cells. Here it maintains histone acetylation by recapturing acetate released by histone deacetylases so it can be re-used for acetylation.

Bulusu et al., 2017, Cell Reports *18*, 647–658
January 17, 2017 © 2017 The Author(s).
http://dx.doi.org/10.1016/j.celrep.2016.12.055

OPEN ACCESS
CellPress

Acetate Recapturing by Nuclear Acetyl-CoA Synthetase 2 Prevents Loss of Histone Acetylation during Oxygen and Serum Limitation

Vinay Bulusu,[1,2] Sergey Tumanov,[1,2] Evdokia Michalopoulou,[1,2] Niels J. van den Broek,[1] Gillian MacKay,[1] Colin Nixon,[1] Sandeep Dhayade,[1] Zachary T. Schug,[1,3] Johan Vande Voorde,[1] Karen Blyth,[1] Eyal Gottlieb,[1] Alexei Vazquez,[1] and Jurre J. Kamphorst[1,2,4,*]
[1]Cancer Research UK Beatson Institute, Garscube Estate, Switchback Road, Glasgow G61 1BD, UK
[2]Institute of Cancer Sciences, University of Glasgow, Garscube Estate, Switchback Road, Glasgow G61 1QH, UK
[3]Present address: The Wistar Institute, 3601 Spruce Street, Philadelphia, PA 19104, USA
[4]Lead Contact
*Correspondence: jurre.kamphorst@glasgow.ac.uk
http://dx.doi.org/10.1016/j.celrep.2016.12.055

SUMMARY

Acetyl-CoA is a key metabolic intermediate with an important role in transcriptional regulation. The nuclear-cytosolic acetyl-CoA synthetase 2 (ACSS2) was found to sustain the growth of hypoxic tumor cells. It generates acetyl-CoA from acetate, but exactly which pathways it supports is not fully understood. Here, quantitative analysis of acetate metabolism reveals that ACSS2 fulfills distinct functions depending on its cellular location. Exogenous acetate uptake is controlled by expression of both ACSS2 and the mitochondrial ACSS1, and ACSS2 supports lipogenesis. The mitochondrial and lipogenic demand for two-carbon acetyl units considerably exceeds the uptake of exogenous acetate, leaving it to only sparingly contribute to histone acetylation. Surprisingly, oxygen and serum limitation increase nuclear localization of ACSS2. We find that nuclear ACSS2 recaptures acetate released from histone deacetylation for recycling by histone acetyltransferases. Our work provides evidence for limited equilibration between nuclear and cytosolic acetyl-CoA and demonstrates that ACSS2 retains acetate to maintain histone acetylation.

INTRODUCTION

Cancer is a disease of unrestrained cell proliferation. This necessitates the continued production of cellular components, including lipid membranes, for the generation of daughter cells (DeBerardinis et al., 2008). The bulk elements of membranes are fatty acids, and their de novo synthesis by cancer cells places a high demand on the precursor acetyl-coenzyme A (AcCoA) (Menendez and Lupu, 2007; Currie et al., 2013). In addition to its role in biomass production, the central position of AcCoA in both anabolic and catabolic pathways makes it a rheostat of the metabolic state of the cell (Pietrocola et al., 2015; Shi and Tu, 2015; Fan et al., 2015). AcCoA abundance directly affects the metabolic activity of many enzymes, and acetylation events on histones and other proteins control cellular functions at the transcriptional and post-translational levels. Disrupting acetylation homeostasis by inhibiting deacetylases (i.e., histone deacetylases [HDACs] and sirtuins) was shown to influence cancer cell survival (Falkenberg and Johnstone, 2014). It is therefore crucial for cells to maintain AcCoA homeostasis.

In solid tumors, cells frequently experience reduced oxygen availability because of aberrant vascularization (Brown and Wilson, 2004). Although, in oxygenated cells, most AcCoA is made from glucose, during hypoxia, stabilization of HIF1 causes cancer cells to shunt most glucose-derived carbon toward lactate (Kim et al., 2006; Papandreou et al., 2006). This diminishes AcCoA production from glucose, and how cancer cells adapt has been a topic of interest. Tracing experiments revealed that glutamine, through reductive carboxylation, is a precursor for AcCoA that is used for fatty acid biosynthesis, also known as lipogenic AcCoA (Wise et al., 2011; Metallo et al., 2011; Mullen et al., 2011). It has later been argued that a reduction in citrate levels in hypoxic cells causes increased reversibility with α-ketoglutarate. Because of this, at least some fatty acid labeling from glutamine could be explained as isotope exchange (i.e., mixing of label) rather than a net reductive flux (Fan et al., 2013).

More recently, it was found that ^{13}C-acetate significantly labels the lipogenic AcCoA pool of hypoxic cells (Kamphorst et al., 2014) and, consequently, lipids (Schug et al., 2015), suggesting that acetate can act as an alternative carbon source for biomass production under these conditions. In a separate study, glioblastomas and brain metastases were shown to oxidize acetate for energy production, indicating that the metabolic fate may depend on tumor type (Mashimo et al., 2014). Importantly, silencing the nuclear-cytosolic isoform of AcCoA synthetase (ACSS2), which converts acetate to AcCoA, reduced tumor growth in a xenograft model of breast cancer (Schug et al., 2015). Furthermore, *ACSS2* deletion diminished tumor burden in a genetically engineered mouse model of hepatocellular carcinoma (Comerford et al., 2014).

This is an open access article under the CC BY license (http://creativecommons.org/licenses/by/4.0/).
CELREP 3443

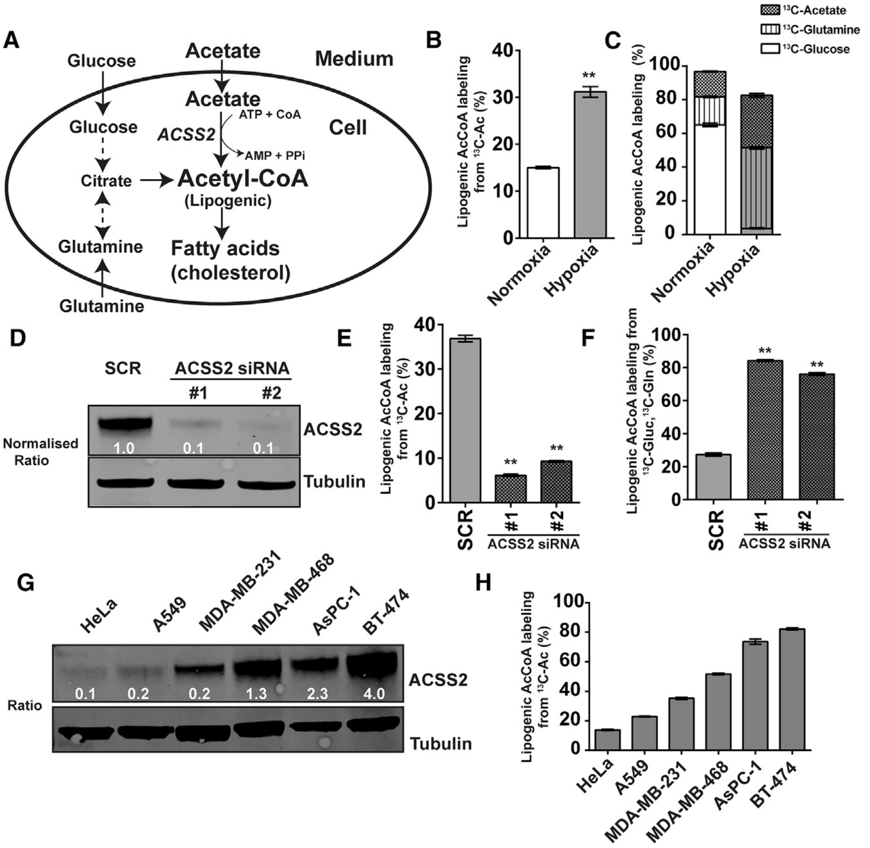

Figure 1. ACSS2 Controls Acetate Incorporation Into Lipogenic Acetyl-CoA

(A) Schematic of lipogenic AcCoA production.

(B) Steady-state ^{13}C labeling (percent) of lipogenic AcCoA from 90 μM U-^{13}C-acetate (Ac) in normoxia or hypoxia (1% O_2).

(C) Steady-state ^{13}C labeling (percent) of lipogenic AcCoA from U-^{13}C-glucose (Gluc), U-^{13}C-glutamine (Gln), and 90 μM U-^{13}C-acetate.

(D) Western blot of ACSS2 from cells transfected with scrambled RNA (SCR) or two independent ACSS2 siRNAs. The normalized ratio is relative to the SCR control.

(E) Steady-state ^{13}C labeling (percent) of lipogenic AcCoA from 90 μM U-^{13}C-Ac in hypoxic SCR or ACSS2 siRNA-treated cells.

(F) Steady-state ^{13}C labeling (percent) of lipogenic AcCoA from U-^{13}C-Gluc and U-^{13}C-Gln in hypoxic SCR or ACSS2 siRNA-treated cells. The medium contained 90 μM ^{12}C-acetate.

(G) ACSS2 western blot from multiple human cancer cell lines under hypoxia (48 hr). Tubulin was used as the loading control.

(H) Steady-state ^{13}C labeling (percent) of lipogenic AcCoA from 500 μM U-^{13}C-Acetate.

(B–F) Experiments were done in MDA-MB-468 cells.

All data are mean ± SD (n = 3); *$p < 0.05$, **$p < 0.01$. See also Figure S1.

The extensive fatty acid labeling from ^{13}C-acetate in hypoxic cancer cells may indicate that the increased ACSS2 expression supports biomass production. It is important to note, however, that the actual carbon contribution to fatty acid synthesis remains to be determined. As with glutamine, the fractional labeling of AcCoA from acetate may not reflect net synthesis because of isotope exchange (Fan et al., 2013). A rapid equilibration between the acetate and AcCoA pools could occur as a consequence of a fast protein acetylation-deacetylation cycle, with the cellular acetate pool also exchanging with labeled medium acetate. This would result in pronounced labeling of AcCoA and, subsequently, fatty acids from ^{13}C-acetate without a net carbon transfer. Therefore, a more extensive evaluation to quantify the contribution of acetate to biomass production is needed. Furthermore, it has been reported that a significant proportion of ACSS2 localizes to the nuclei of tumor cells (Comerford et al., 2014), and how much exogenous acetate can contribute to nuclear processes such as histone acetylation remains unknown.

Here we apply innovative stable isotope tracing and mass spectrometry approaches to quantify acetate consumption and utilization by downstream pathways in a panel of cancer cell lines with varying levels of ACSS2 expression. We find that the combined expression of ACSS1 and ACSS2 determines the net acetate uptake rate. Exogenous acetate is used extensively by the mitochondria and for lipogenesis, and the demand for acetate substantially exceeds its uptake. As a consequence, exogenous acetate only modestly labels histone-bound acetate. However, nuclear localization of ACSS2 increases during oxygen and serum limitation, and nuclear ACSS2 is prominent in poorly perfused, hypoxic tumor regions of a mouse model of breast cancer. We find that the primary function of nuclear ACSS2 is to retain endogenous acetate released by deacetylases to maintain histone acetylation and propose that this is especially relevant in hypoxic and nutrient-limited areas of the tumor.

RESULTS

ACSS2 Expression Dictates Lipogenic AcCoA Labeling from U-^{13}C-Acetate in Hypoxic Cancer Cells

A substantial fraction of the AcCoA used for fatty acid biosynthesis (i.e., lipogenic AcCoA) is produced from glucose and glutamine (Figure 1A). Alternatively, lipogenic AcCoA can be produced from acetate by ACSS2, and this pathway has been reported to be induced in hypoxic tumor cells (Schug et al., 2015; Comerford et al., 2014). Importantly, we determine the contribution of these different precursors to lipogenic AcCoA from fatty acid labeling without the need to analyze AcCoA directly, which is a mixture of all pools in the cell (Kamphorst et al., 2014; Tumanov et al., 2015).

We previously reported a substantial contribution of acetate to lipogenic AcCoA in hypoxic cancer cells, including MDA-MB-468 breast cancer cells (Kamphorst et al., 2014). Because the concentration of acetate in human plasma is 50–250 μM (Schug et al., 2015), we sought to confirm the propensity of these cells to use acetate in the lower end of this physiological range. Normoxic and hypoxic (1% O_2) MDA-MB-468 cells were exposed

to 90 μM U-^{13}C-acetate. Although a relatively small fraction of lipogenic AcCoA labeled from U-^{13}C-acetate in normoxic cells, this increased to approximately 30% in hypoxia (Figure 1B). Next, cells were cultured in the presence of U-^{13}C-glucose, U-^{13}C-glutamine, and U-^{13}C-acetate to confirm that, combined, these are the major substrates for lipogenic AcCoA production (Figure 1C). As expected, the contribution from glucose dropped considerably in hypoxia, and labeling from both U-^{13}C-glutamine and U-^{13}C-acetate increased. Under both conditions, the majority of lipogenic AcCoA was labeled from all substrates combined. The somewhat lower labeling under hypoxic conditions could, in part, be explained by the presence of residual unlabeled acetate (~15 μM) in the medium. Therefore, when provided in a physiologically relevant concentration, acetate is an important substrate for lipogenic AcCoA.

ACSS2 is reportedly responsible for acetate utilization for lipogenesis, but an accurate quantitation of its contribution is lacking. When silencing ACSS2 expression in MDA-MB-468 cells using small interfering RNA (siRNA) (Figure 1D), we observed a pronounced reduction in labeling of lipogenic AcCoA from U-^{13}C-acetate (Figure 1E). Similar results were obtained for BT-474 cells (Figures S1A and S1B). Under these conditions, labeling from U-^{13}C-glucose and U-^{13}C-glutamine increased to ~80% (Figure 1F). We found that unlabeled acetate in the medium suppressed labeling of lipogenic AcCoA from U-^{13}C-glucose and U-^{13}C-glutamine, indicating that acetate is preferentially used for lipogenesis under these conditions (Figure S1C). The increased labeling from U-^{13}C-glucose and U-^{13}C-glutamine upon ACSS2 knockdown may not indicate increased AcCoA biosynthesis from these substrates; net production may remain equal while fractional labeling increases because of the drop in the contribution from acetate. This supports earlier findings that acetate, glucose, and glutamine are the main substrates for lipogenic AcCoA (Kamphorst et al., 2014).

To further investigate the role of ACSS2 expression in acetate utilization for lipogenic AcCoA, we performed U-^{13}C-acetate tracing in a set of six cancer cell lines originating from cervical (HeLa), lung (A549), pancreatic (AsPC-1), and breast tumors (MDA-MB-231, MDA-MB-468, and BT-474). This set covers a wide range of ACSS2 expression and facilitates assessment of the relation between ACSS2 expression and lipogenic AcCoA labeling in cells with the same or different tissue backgrounds (Figures 1G and 1H). Across cell lines, a clear correlation was found between ACSS2 expression and lipogenic AcCoA labeling from U-^{13}C-acetate. ACSS1 expression generally appeared to be low in most cell lines, except for BT-474 cells (Figure S1D). In this cell line, silencing ACSS1 had no effect on ACSS2 expression but did cause a small increase in lipogenic AcCoA labeling from U-^{13}C-acetate (Figures S1E and S1F), perhaps because of some degree of competition between ACSS1 and ACSS2 for the available acetate. Together, these results show a strong correlation between ACSS2 expression and lipogenic AcCoA labeling from U-^{13}C-acetate.

Cancer Cells Exchange Acetate with the Medium, and Net Exchange Is Determined by ACSS2 Expression

As stated earlier, fatty acid labeling from U-^{13}C-acetate is not sufficient to determine the carbon contribution from acetate because of possible cycling and exchange (Fan et al., 2013; Kamphorst et al., 2014). To measure acetate directly and quantify its net uptake by cancer cells, we developed a method based on alkylation of acetate to its propyl ester derivative using a methyl chloroformate (MCF)-catalyzed derivatization reaction and subsequent gas chromatography-mass spectrometry (GC-MS) analysis (Experimental Procedures; Figure S2A; Tumanov et al., 2016). For our analyses, we used $^{2}H_3$-acetate as an internal standard, enabling within-run quantification of both ^{12}C-acetate and U-^{13}C-acetate. We applied this method to analyze uptake of 90 μM U-^{13}C-acetate spiked into the medium by both normoxic and hypoxic MDA-MB-468 cells. The hypoxic cells consumed almost all U-^{13}C-acetate (Figure 2A). Interestingly, although, in normoxic cells, lipogenic AcCoA labeled only marginally from acetate (Figure 1B), U-^{13}C-acetate uptake was comparable with hypoxic cells.

To our surprise, in addition to a reduction in U-^{13}C-acetate because of consumption, unlabeled acetate (^{12}C-acetate) accumulated in the cell-conditioned medium (Figure 2B). We concluded that this acetate was being released by cells because the initial medium ^{12}C-acetate and U-^{13}C-acetate concentrations remained stable when incubated in the absence of cells (Figure S2B). Furthermore, this acetate labeled from U-^{13}C-glucose and U-^{13}C-glutamine (Figure S2C). Acetate release was approximately equal to acetate consumption, resulting in an unchanged total medium acetate concentration (Figure 2C). This was also observed with physiological concentrations of glucose (5.5 mM) and glutamine (0.65 mM) (Figure S2D). Under the conditions described here, lipogenic AcCoA and, hence, fatty acid labeling from U-^{13}C-acetate was prominent (Figure 1B). However, because no net uptake of acetate could be observed, it is caused by cycling between acetate and lipogenic AcCoA and exchange between cellular and medium acetate.

To expand our analysis of acetate uptake (u), release (r), and net exchange (e) flux independently of cell number and growth, we cultured cells in medium containing various concentrations of U-^{13}C-acetate. The net exchange rate was calculated as the change in total medium acetate divided by the area under the cell growth curve. To deconvolute the uptake and release components of the exchange, we used the balance equations for the exchange of total acetate ($e = r - u$) and ^{12}C-acetate ($xe = ar - bu$), where x is the concentration change in medium ^{12}C-acetate relative to the concentration change of total acetate, a is the average ^{12}C-acetate fraction inside cell, and b is the average ^{12}C-acetate fraction in the medium. The parameters e, x, and b can be estimated from the measurement of medium ^{12}C- and U-^{13}C-acetate at different time points (see Supplemental Experimental Procedures for more information). The intracellular free acetate concentration was too low to quantify accurately. We therefore considered all biologically relevant values of the intracellular acetate fractions (Supplemental Experimental Procedures). Under all conditions tested, both uptake and release of acetate were ~3–4 mmol/hr/L cell volume, with little variation because of extracellular acetate concentration or oxygenation (Figure 2D). In contrast, the net exchange rate remained close to zero, with a tendency toward net acetate release.

We expanded our net acetate exchange experiments to the panel of six cancer cell lines (Figure 1G) cultured in hypoxia

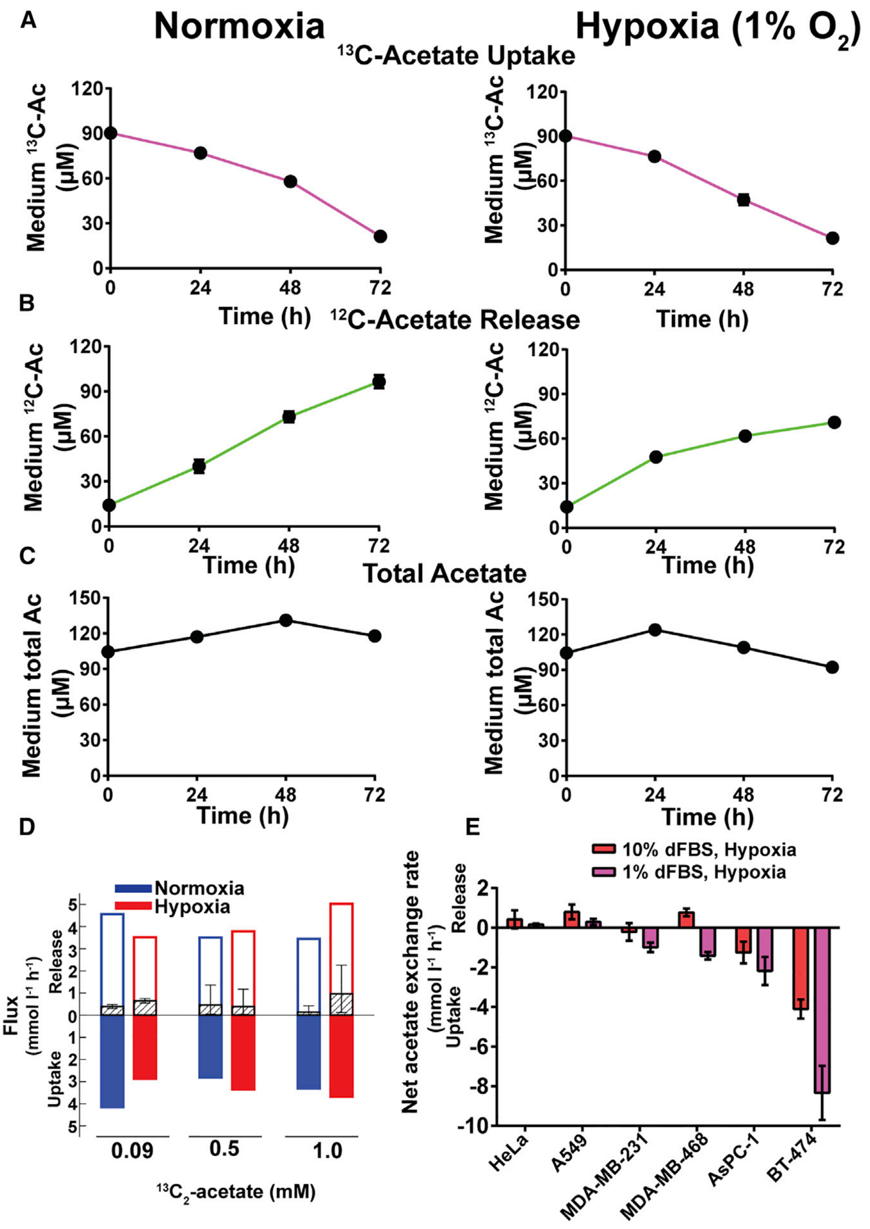

Figure 2. Cancer Cells Take up and Release Acetate, and ACSS2 Expression Dictates Net Exchange

(A) Time course of U-^{13}C-Ac (90 μM) uptake by MDA-MB-468 cells in normoxia and hypoxia.

(B) Time course of unlabeled (^{12}C) Ac release into the medium.

(C) Time course of total Ac (U-^{13}C-Ac + ^{12}C-Ac) concentration in medium.

(D) Estimated acetate release, uptake, and exchange fluxes in normoxic (blue) and hypoxic (red) MDA-MB-468 cells for multiple concentrations of U-^{13}C-Ac in the medium. The open bars represent the 5% quantile of the acetate release flux (i.e., with 95% confidence, the release flux is higher than the bar height). The filled bars represent the 5% quantile of the acetate uptake flux (i.e., with 95% confidence, the uptake flux is higher than the bar height). The dashed bars and error bars represent the median and 95% confidence intervals of the acetate exchange flux.

(E) Net acetate exchange for the panel of cell lines in hypoxia and 10% or 1% dialyzed serum. Cell lines are ordered based on increasing ACSS2 expression from left to right.

All data are mean ± SD (n = 3). See also Figure S2.

and high (10%) or low (1%) serum (Figure 2E). The cell lines with low ACSS2 expression (HeLa and A549) showed a net release of acetate that was reduced in low serum. Generally, culturing cells in low serum and hypoxia caused induction of ACSS2 expression (Figure S2E; Schug et al., 2015), and this led to a net acetate uptake for MDA-MB-231 and MDA-MB-468 cells. For the latter, net exchange was comparable with AsPC-1 cells, which have similar levels of ACSS2. BT-474 cells express high levels of ACSS2 because of gene amplification (Schug et al., 2015). They were found to most avidly take up acetate, especially in low serum (~8 mmol/hr/L cell volume). Together, these results demonstrate that cells exchange acetate with the medium and that high ACSS2 expression promotes net acetate uptake.

Mitochondrial and Lipogenic Utilization of Exogenous Acetate Limits Its Use for Histone Acetylation

Net acetate uptake is highest in low serum, and this may be more reflective of the tumor microenvironment. We therefore used low serum for subsequent analyses. After being activated to AcCoA, exogenous acetate can be directed toward mitochondria for oxidation (Mashimo et al., 2014), used for biomass production (particularly lipogenesis), or used for acetylation. To determine whether acetate feeds into mitochondria, we analyzed tricarboxylic acid (TCA) cycle intermediate labeling from U-^{13}C-acetate. Substantial (15%–25%) labeling occurred in BT-474 cells and, to a lesser extent, in MDA-MB-468 cells despite the hypoxia and low serum (Figures 3A and 3B). ACSS1 is responsible for mitochondrial utilization of acetate, and its knockdown substantially, although not completely, reduced labeling of TCA cycle intermediates (Figures S3A and S3D). Conversely, overexpressing ACSS1 in MDA-MB-468 cells led to an ~4-fold increase in labeling of TCA cycle intermediates (Figures S3B and S3C). In BT-474 cells, ACSS1 silencing caused a concomitant reduction in exogenous acetate uptake by approximately 30% (Figures 3C and 3D). Because ACSS1-generated AcCoA does not appear to feed into fatty acid biosynthesis, it provides an estimate of mitochondrial acetate utilization.

A major AcCoA-consuming pathway in the cytosol is lipogenesis. To determine the AcCoA demand of this pathway,

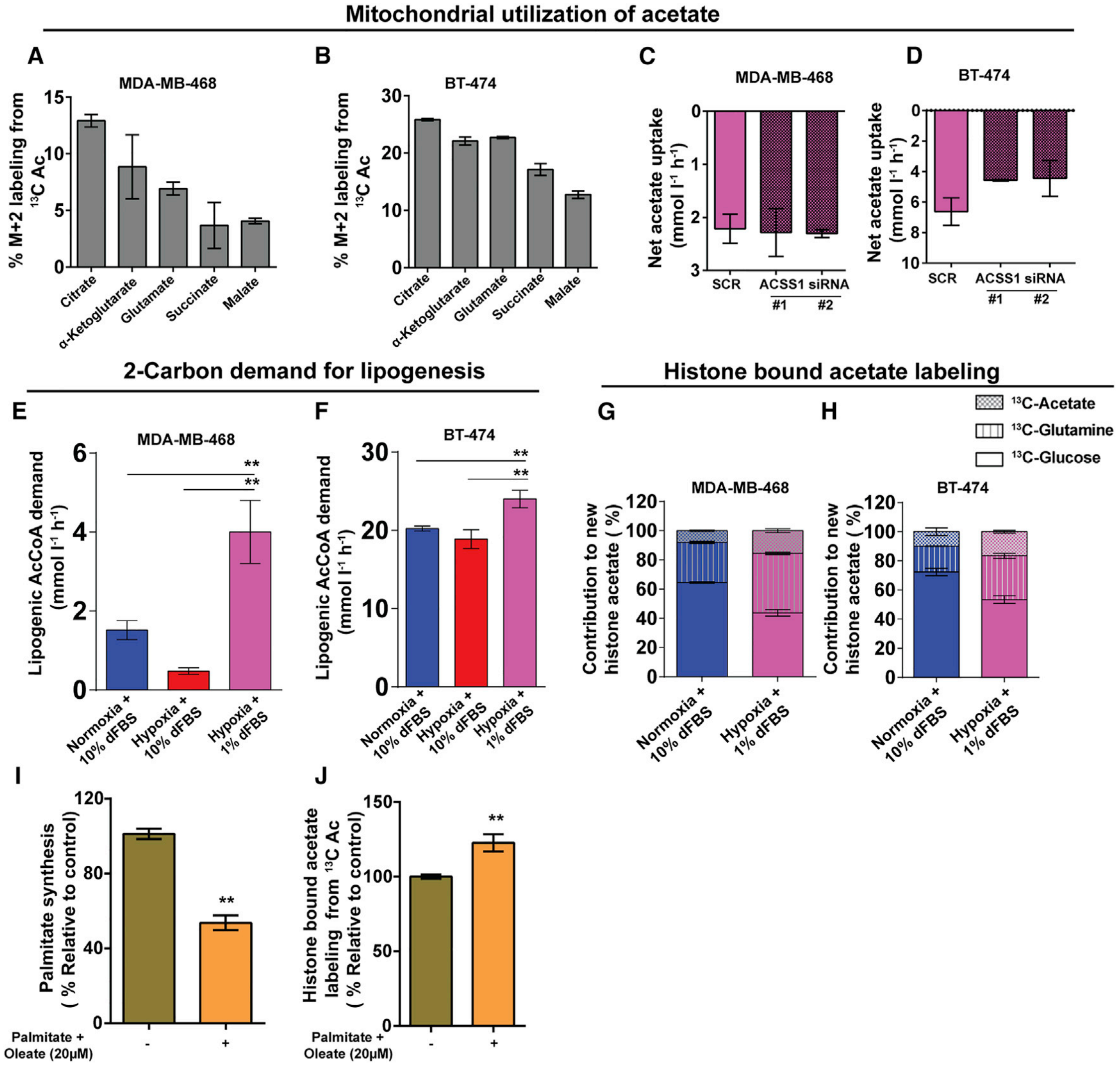

Figure 3. High Mitochondrial and Lipogenic Demand for Acetate Limits Its Use for Histone Acetylation

(A and B) Steady-state labeling of TCA cycle intermediates from U-^{13}C-Ac (500 μM) in (A) MDA-MB-468 cells and (B) BT-474 cells.

(C and D) Effect of ACSS1 knockdown on net acetate uptake in (C) MDA-MB-468 and (D) BT474 cells.

(E and F) Lipogenic AcCoA demand for de novo fatty acid synthesis as determined by kinetic flux profiling (Supplemental Experimental Procedures) for (E) MDA-MB-468 and (F) BT-474 cells.

(G and H) Steady-state labeling of histone-bound acetate from U-^{13}C-Gluc, U-^{13}C-Gln, and U-^{13}C-Ac for (G) MDA-MB-468 and (H) BT-474 cells.

(I) Effect of free fatty acid supplementation on fatty acid biosynthesis in MDA-MB-468 cells.

(J) Effect of free fatty acid supplementation on steady-state, histone-bound acetate labeling from U-^{13}C-Ac in MDA-MB-468 cells.

For (A)–(D), (I), and (J), data are from cells in hypoxia (1% O_2) and low serum (1%).

All data are mean ± SD (n = 3); *$p < 0.05$, **$p < 0.01$. See also Figure S3.

we performed kinetic flux profiling by analyzing the incorporation of ^{13}C from U-^{13}C-glucose, U-^{13}C-glutamine, and U-^{13}C-acetate into the palmitate pool over time (Supplemental Experimental Procedures; Figures 3E and 3F; Tumanov et al., 2015). The time-based label incorporation reflects the synthesis rate, which can be deduced by fitting a differential equation (Yuan et al., 2008). In agreement with previous reports, hypoxia caused cells to reduce palmitate synthesis rates in the presence of high serum, particularly in MDA-MB-468 cells, because of the presence of exogenous lipids (Kamphorst et al., 2013; Young et al., 2013). Exposing cells to low serum led to a potent induction of the palmitate synthesis rate, increasing the demand for lipogenic AcCoA. Strikingly, in low oxygen and serum, the two-carbon acetyl demand for palmitate synthesis alone outstripped the exogenous acetate uptake rate ~2- to 3-fold. The exact flux of acetate into lipogenesis could not be determined because of the exchange. However, labeling of lipogenic AcCoA from U-^{13}C-acetate is profound (Figure 1H), and exogenous acetate suppresses labeling from U-^{13}C-glucose and U-^{13}C-glutamine (Figure S1C). It is therefore likely that a

large proportion of the exogenous acetate is consumed for lipogenesis.

Last, we determined histone-bound acetate labeling from U-^{13}C-glucose, U-^{13}C-glutamine, or U-^{13}C-acetate. We did this by hydrolyzing acetate from purified histones rather than measuring individual acetylated peptides (Experimental Procedures). Therefore, the measured labeling reflects the aggregate of the entire histone-bound acetate pool. Not surprisingly, glucose was the major carbon donor under normoxic conditions (Figures 3G and 3H). In hypoxia and low serum, the contribution from glucose dropped, and the percent labeling from U-^{13}C-glutamine increased ~2-fold, indicating that reductive carboxylation also occurs for histone acetylation (Figures 3G and 3H). Acetate was the smallest contributor in both cell lines (<10%). Although hypoxia and low serum caused this to increase to ~15%, it was still much less than either glucose and glutamine, and multiple-fold less than for lipogenic AcCoA (Figure 1H; Figure S3F). ACSS1 overexpression in MDA-MB-468 cells did not affect this (Figure S3E). Thus, exogenous acetate is inefficiently used for histone acetylation.

We hypothesized that, upon cell entry, exogenous acetate would be rapidly activated to AcCoA before being able to reach the nucleus and that this AcCoA would locally feed into the carbon-demanding pathways of mitochondrial metabolism and lipogenesis rather than being used for histone acetylation. We therefore supplied the free fatty acids palmitate and oleate to cells to reduce lipogenic demand for AcCoA. Free fatty acids are avidly consumed by cells (Kamphorst et al., 2013), and this led to an ~50% reduction in de novo lipogenesis (Figure 3I). This was accompanied by a significant increase in labeling of histone-bound acetate from the exogenously supplied U-^{13}C-acetate (Figure 3J). Thus, the high lipogenic consumption of exogenous acetate at least partly explains its limited use for histone acetylation.

Oxygen and Serum Limitation Promote Nuclear Localization of ACSS2, and ACSS2 Is Prominently Nuclear in Hypoxic Tumor Regions

Although exogenous acetate is only sparingly used for histone acetylation, ACSS2 has been reported to be substantially nuclear (Comerford et al., 2014). To quantify this, we performed an analysis of ACSS2 localization in MDA-MB-468 cells using immunofluorescence (Figures 4A and 4B). Under normoxic and high serum conditions, ~15% of the ACSS2 pool was localized to the nucleus. However, exposing the cells to low serum and oxygen caused a significant, ~3-fold increase in the fraction of nuclear ACSS2 (Figure 4B). A similar trend was observed for BT-474 cells (Figure S4). Further assessment of the individual factors indicated that both serum and oxygen limitation contribute to the increased nuclear localization of ACSS2, although the effect of serum limitation appeared to be strongest (Figure 4B). Low glucose did increase the localization of ACSS2 to the nucleus in normoxic cells but not in hypoxic cells.

To determine the in vivo expression and localization of ACSS2 as a function of oxygenation, serial sections were prepared from established tumors of the mouse mammary tumor virus polyoma middle T antigen (MMTV-PyMT) genetically engineered mouse model of breast cancer (Guy et al., 1992). These were immunostained for ACSS2, the hypoxic marker carbonic anhydrase IX (CAIX), and the blood vessel marker CD31, in addition to H&E staining (Figure 4C). Overall, ACSS2 staining was particularly intense in hypoxic (high CAIX, low CD31) regions of the tumor and exhibited prominent nuclear localization (Figure 4C). Indeed, scoring of nuclear intensity of ACSS2 staining revealed that nuclear ACSS2 levels are substantially higher in hypoxic regions than in perfused, normoxic regions (Figure 4D). These data show that cultured cells in low oxygen and serum more strongly resemble the conditions of the tumor with respect to ACSS2 localization. Additionally, it points to an important role for ACSS2 in the nuclei of hypoxic tumor cells.

ACSS2 Recaptures Endogenously Produced Acetate

We considered whether the primary function of nuclear ACSS2 might be to recapture acetate released by histone deacetylation. In support of an acetate-recapturing function of ACSS2, we noticed that cells that avidly consumed U-^{13}C-acetate released relatively little ^{12}C-acetate (Figure S5A), arguing that ACSS2 captures exogenous acetate but also prevents the release of endogenously produced acetate by recapturing it. To quantify endogenous acetate release independently of cell number, we plotted the ratio of ^{12}C-acetate released versus U-^{13}C-acetate consumed in relation to ACSS2 expression for the six cell lines in our panel. We found a strong inverse correlation between this ratio and ACSS2 expression (Figure 5A), indicating that high ACSS2 expression not only facilitates uptake of exogenous acetate but also efficient recapturing of endogenously produced acetate.

To further investigate the role of ACSS2 in recapturing endogenously produced acetate, we silenced its expression in MDA-MB-468 cells by siRNA. In agreement with ACSS2 mediating uptake of exogenous acetate, this largely abrogated U-^{13}C-acetate consumption (Figure 5B). Consistent with the recapturing hypothesis, ^{12}C-acetate release markedly increased upon ACSS2 knockdown (Figure 5C). Under this serum- and oxygen-limited condition, the net result was a stagnant total acetate level (Figure 5D). Further demonstrating that ACSS2 determines ^{12}C-acetate release/U-^{13}C-acetate uptake, this ratio increased upon ACSS2 knockdown in MDA-MB-468 and BT-474 cells (Figures 5E and 5F). A similar trend was observed in hypoxia and 10% serum (Figures S5B and S5C). To determine whether ACSS1 recaptures endogenous acetate, we silenced its expression in BT-474 cells. This also led to increased ^{12}C-acetate release-to-U-^{13}C-acetate uptake ratio (Figure S5D). This demonstrates that, in addition to taking up exogenous acetate, ACSS2 and ACSS1 function to recapture endogenously produced acetate. Net acetate uptake results from a combined intake of exogenous acetate and retention of intracellular acetate.

ACSS2 Maintains Histone Acetylation during Oxygen and Serum Limitation

The acetate re-capturing function of ACSS2 and its prominent nuclear localization during oxygen and serum limitation prompted us to test whether ACSS2 maintains histone acetylation. This could be important because histones are among the most abundant proteins in mammalian cells and are heavily acetylated. With a residence half-life of only minutes, turnover of most histone acetylation marks is quite rapid (Evertts et al., 2013). Under oxygen- and serum-replete conditions, silencing

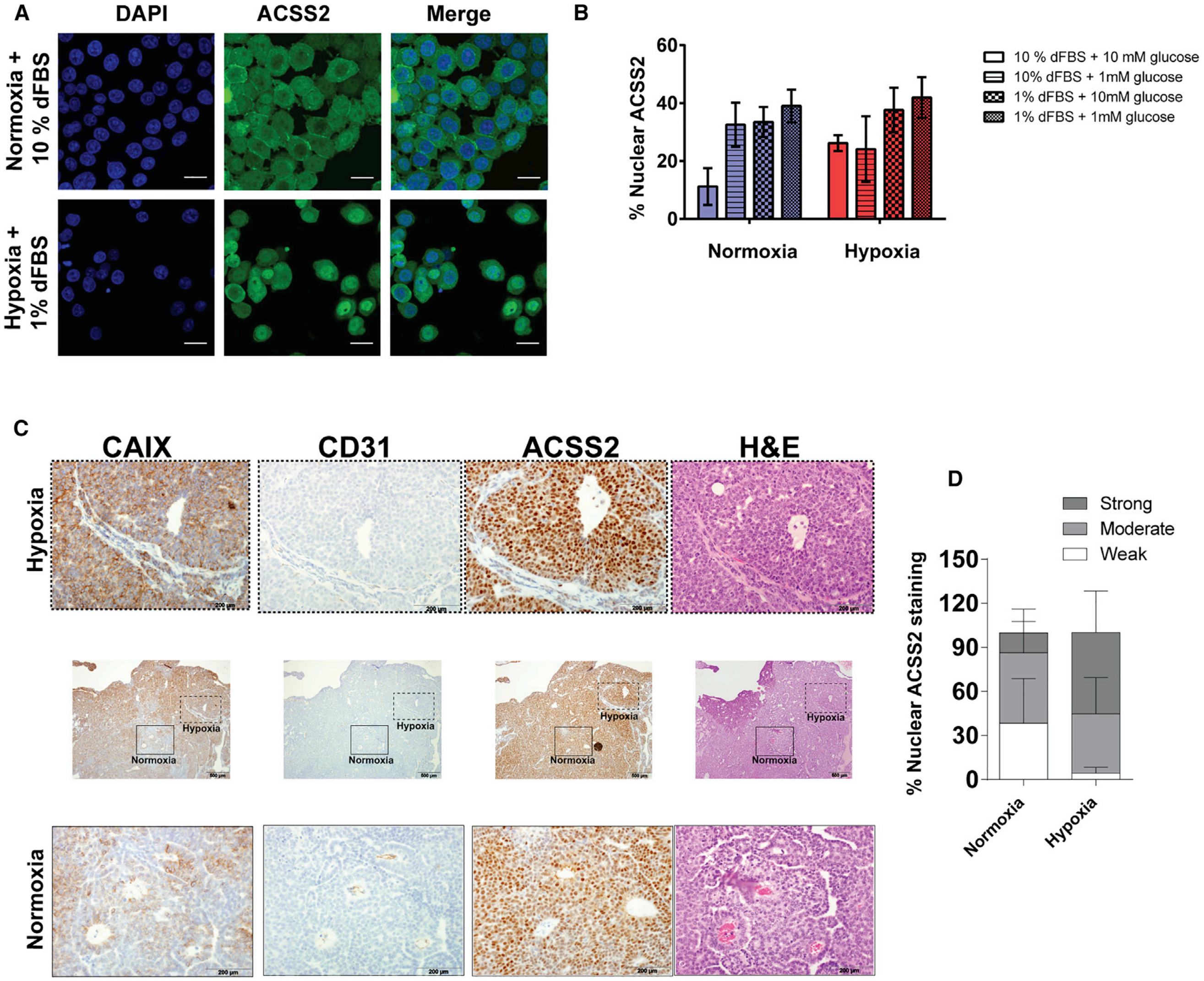

Figure 4. ACSS2 Localizes to the Nucleus during Oxygen and Serum Limitation and Is Prominently Nuclear in Tumors

(A) Representative images of DAPI (nuclear) and ACSS2 staining (separately and merged) in MDA-MB-468 cells in normoxia and 10% serum or hypoxia and 1% serum. Scale bar, 20 μm.

(B) Quantification of the nuclear fraction of ACSS2 (percent) in MDA-MB-468 cells cultured under different conditions. Data are mean ± SD of three independent experiments (seven or more images per experiment).

(C) Immunohistochemical staining of serial sections from a representative tumor of the MMTV-PyMT mouse model for carbonic anhydrase 9 (CAIX, a hypoxic marker), CD31 (a marker for blood vessels), ACSS2, and H&E staining.

(D) Scoring of ACSS2 nuclear intensity in normoxic and hypoxic tumor regions. Ten different ROIs were selected for normoxic and hypoxic regions of two tumors (five ROIs each), and the percentages of cells with weak, moderate or strong nuclear ACSS2 staining were scored. Data are mean ± SD (n = 10 ROIs).

See also Figure S4.

ACSS2 did not result in a decrease but, rather, a modest increase in acetylation of histones H3 (AcH3) and H4 (AcH4) (Figures 6A and 6B), which may be due to HDAC inhibition by nuclear acetate accumulation. In contrast, when silencing ACSS2 in low oxygen and serum, a significant decrease was observed in the acetylation of both H3 and H4. No acetate was added to the medium in this experiment, further reinforcing the notion that ACSS2 maintains histone acetylation by acetate recapturing.

We next asked whether any of the acetate released into the medium originated from histone deacetylation. If so, then inhibition of HDACs should lead to a drop in acetate release. We evaluated this with a short incubation protocol (6–8 hr depending on the cell line). Incubation of MDA-MB-468 cells with the pan-HDAC inhibitor panobinostat led to a significant reduction in acetate release into the medium of the control (scramble [SCR]) MDA-MB-468 and BT-474 cells (Figures 6C and 6D; Figure S6A). Comparable results were obtained with the HDAC inhibitor sodium butyrate (Figure S6B). As expected, silencing of ACSS2 resulted in increased release of acetate. Importantly, treatment with panobinostat in ACSS2-silenced cells caused a greater

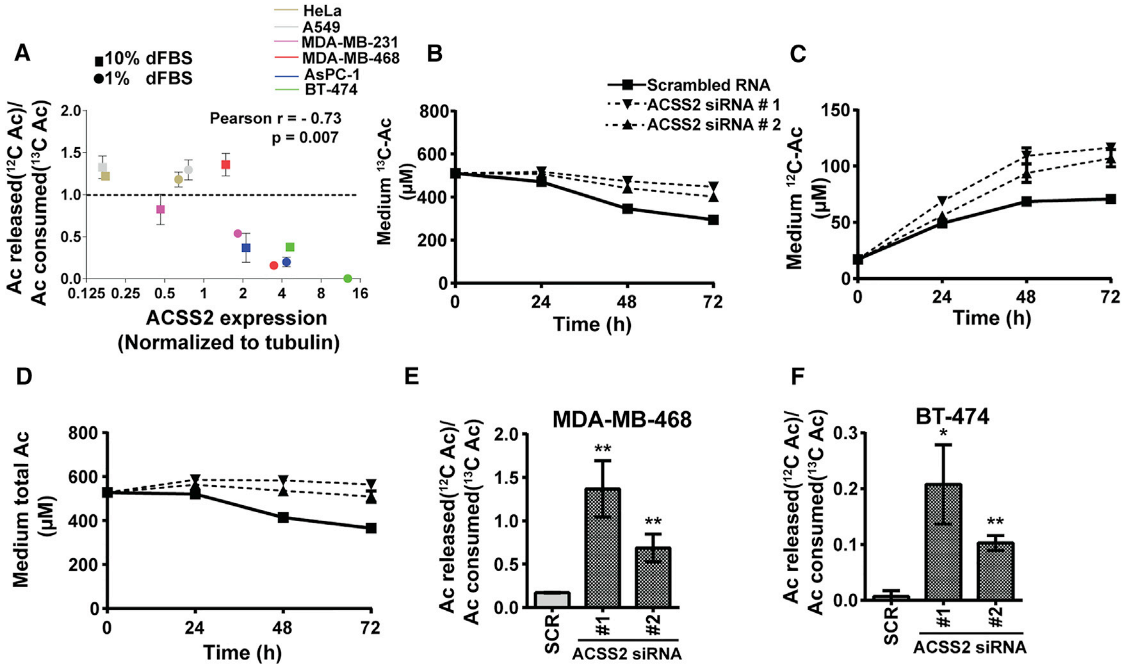

Figure 5. ACSS2 Recaptures Endogenously Produced Acetate

(A) Plot of the ratio of ^{12}C-acetate released/^{13}C-acetate consumed to ACSS2 expression in cancer cells in hypoxia and 10% or 1% dialyzed serum. Statistics are from Pearson's correlation analysis using GraphPad Prism software.

(B) Medium ^{13}C-acetate consumption by siRNA-treated MDA-MB-468 cells exposed to hypoxia and 1% dialyzed serum.

(C) Medium ^{12}C-acetate production in the same experiment.

(D) Total acetate in medium of the same experiment.

(E) Ratio of ^{12}C-acetate released to/ ^{13}C-acetate consumed by MDA-MB-468 cells treated with SCR or ACSS2 siRNAs.

(F) The same for BT-474 cells.

All data are mean ± SD (n = 3); $^{*}p < 0.05$, $^{**}p < 0.01$. See also Figure S5.

absolute reduction in acetate release compared with SCR cells, giving further credence to the acetate recapturing function of ACSS2. We additionally tested the involvement of other enzymes in acetate release. Incubation with the sirtuin inhibitors sirtinol (Figure S6C) and nicotinamide (Figure S6D) led to a maximum ~30% reduction in acetate release. In contrast, knockdown of amino acid deacetylases aminoacylase 1 (ACY1) and ACY3, which deacetylate free acetylated amino acids, and aspartoacylase (ASPA), which deacetylates N-acetyl aspartate, did not noticeably affect acetate release (Figure S6E). Thus, acetate release occurs as a consequence of histone deacetylation reactions, and acetate recapturing by ACSS2 maintains histone acetylation during limited oxygen and serum availability.

DISCUSSION

ACSS2 was recently found to be important for tumor growth (Schug et al., 2015; Comerford et al., 2014; Mashimo et al., 2014). However, a quantitative understanding of how it controls acetate exchange and supports downstream metabolic processes is still limited. Here we used innovative stable isotope tracing approaches to interrogate acetate metabolism in cancer cells. One of the findings is that cellular acetate freely exchanges with the medium and that net acetate uptake is controlled by ACSS2 expression. ACSS2 appears to act in a manner similar to hexokinase. Just like hexokinase phosphorylates glucose to effectively trap it inside the cell to commit it to downstream metabolism, acetate is captured by ACSS2 and "primed" for metabolic use. In addition to taking up exogenous acetate, our results demonstrate that ACSS2 recaptures acetate released by deacetylation processes to retain it in the cell. It is the combination of these two activities that determines the net acetate uptake rate.

An important aspect of our work is the determination of acetate utilization fluxes. For example, we were able to estimate mitochondrial utilization from the reduction in net acetate uptake upon ACSS1 silencing. This revealed that approximately one-third of the consumed acetate is dedicated for mitochondrial use in BT-474 cells, presumably mostly for oxidation but, perhaps, also for mitochondrial protein acetylation (Baeza et al., 2016). A major consumer of acetate carbon in the cytosol is fatty acid biosynthesis. We find that, in high serum, hypoxia lowers fatty acid biosynthesis, in line with earlier observations (Kamphorst et al., 2013; Young et al., 2013). In hypoxia and low serum, on the other hand, despite reduced growth of both MDA-MB-468 and BT-474 cells, fatty acid biosynthesis is significantly elevated. (Figures 3E and 3F). This may explain the increased efficiency of lipogenic inhibitors under these conditions (Schug et al., 2015; Peck et al., 2016). In both cell lines, the demand for lipogenic AcCoA alone exceeds acetate uptake by at least 2-fold. The cycling between acetate and acetyl-CoA

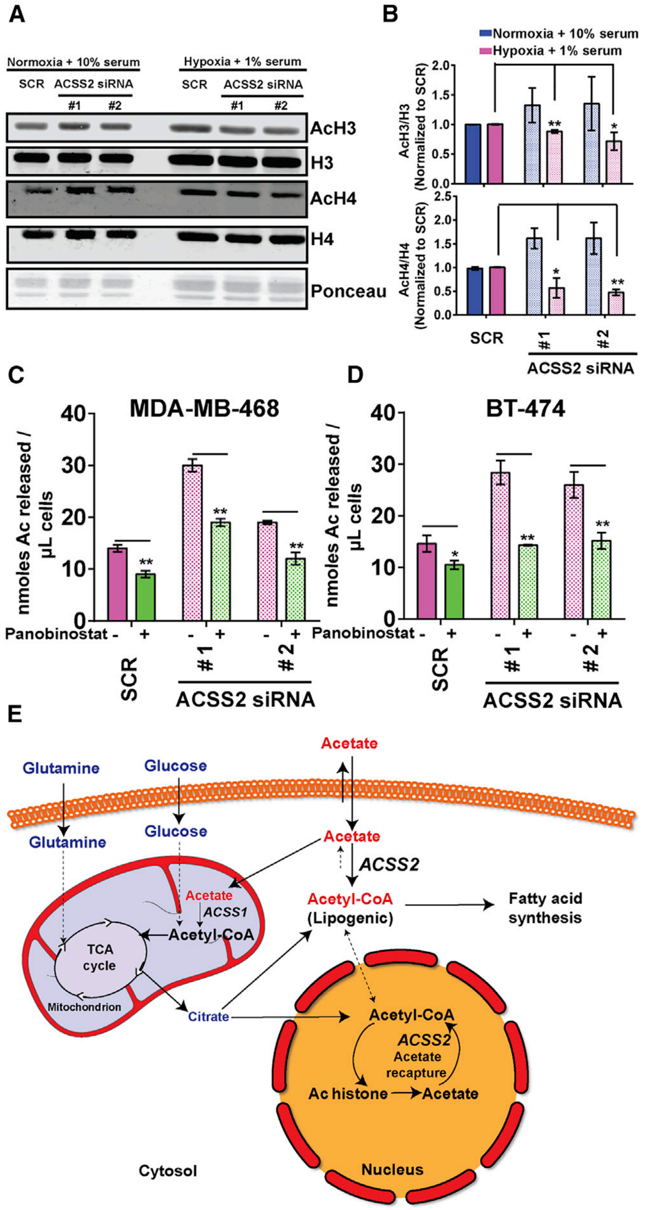

Figure 6. ACSS2 Recaptures Acetate Released from Histone Deacetylation

(A) Western blot of AcH3 and AcH4 in MDA-MB-468 cells upon transfection with SCR or ACSS2 siRNAs. Cells were cultured under the indicated conditions for 48 hr after transfection under either condition.

(B) Bar plot of the same data, with AcH3 normalized to histone H3 and AcH4 to histone H4, with normalized values expressed relative to the scrambled RNA.

(C) Acetate release by MDA-MB-468 cells transfected with SCR or ACSS2 siRNAs and with or without 50 µM panobinostat for 6 hr in low oxygen and serum.

(D) The same for BT-474 cells but with 8-hr incubation.

(E) Schematic of acetate metabolism in oxygen- and serum-limited cancer cells.

For (B)–(D), data are mean ± SD (n = 3); $^{*}p < 0.05$, $^{**}p < 0.01$.

See also Figure S6.

and exchange of intracellular acetate with the medium prohibits accurate quantification of the flux from acetate into fatty acids (Fan et al., 2013), but given the avid and preferential labeling of lipogenic AcCoA from acetate (Figure 1H; Figure S1C), it likely consumes most of the acetate taken up.

To investigate the use of exogenous acetate in the nucleus, we analyzed labeling of histone-bound acetate. This labeling is representative of the aggregate of the histone acetate pool because we measure total (tail and core) histone-bound acetate by subjecting isolated histones to hydrolysis to release bound acetate (Tumanov et al., 2016). This approach enables comparison of the nuclear acetate pool versus other pools in the cells in terms of gross fluxes and pool sizes. As expected, histone-bound acetate labeled substantially from U-^{13}C-glucose and, to a lesser extent, from U-^{13}C-glutamine under normoxic conditions (Everts et al., 2013; Lee et al., 2014). The relative contribution from U-^{13}C-glutamine increased in hypoxia, showing that reductive carboxylation in addition to fatty acids also affects histone acetylation. In line with our observation that the combined mitochondrial and lipogenic two-carbon demand exceeds uptake of exogenous acetate, we did not observe substantial labeling from U-^{13}C-acetate under either normoxic or hypoxic conditions and certainly less than what is observed for lipogenic AcCoA. This agrees with a recent study demonstrating the inefficient use of acetate-derived AcCoA for histone acetylation (Zhao et al., 2016). Supplementing free fatty acids to reduce lipogenesis increased histone-bound acetate labeling from exogenous U-^{13}C-acetate (Figures 3I and 3J). An explanation for our observations is that the majority of exogenous acetate is activated to AcCoA, either by the mitochondrial ACSS1 or the cytosolic ACSS2, before it can reach the nucleus. Because of the high demand of AcCoA-consuming pathways, especially lipogenesis, much of the produced AcCoA is used before it can find its way into the nucleus.

The fact that we can measure overall histone-bound acetate labeling and that exogenous acetate is preferentially used for lipogenesis relative to nuclear histone acetylation provides a fundamental insight into metabolic compartmentalization. Histone-bound acetate can be regarded as a proxy for nuclear AcCoA, and lipogenic AcCoA is representative of the cytosolic AcCoA pool. It is thought that metabolites, including AcCoA, in the cytosol and nucleus freely equilibrate. If this occurs on a large scale, this would result in identical labeling patterns of the cytosolic and nuclear AcCoA pools. However, we observe striking differences in their steady-state labeling patterns, indicating that exchange is fairly limited and that the pools are mostly maintained separately. This gives further credence to the notion that local consumption of AcCoA is too rapid to allow extensive exchange between AcCoA in the separate compartments to occur.

An important finding of this work is that the nuclear localization of ACSS2, which increases in low oxygen and serum, prevents loss of histone deacetylation. We find that ACSS2 staining is prominent in tumors of the MMTV-PyMT model of breast cancer and that the intensity of nuclear staining increases in the hypoxic regions of the tumor (Figures 4C and 4D). Given that cells in these regions also likely experience low nutrient availability, it is plausible that retention of acetyl units by ACSS2 to maintain histone acetylation is a relevant mechanism in vivo. Although the depth

of metabolic analysis will be less in in vivo tracing experiments, and comparison of perfused and hypoxic regions is far from trivial, it may be valuable to further explore certain aspects of acetate metabolism. For example, prolonged infusion with ^{13}C-acetate may help to assess the propensity to incorporate the label as a function of ACCS2 expression. Additionally, to investigate acetate recapturing by ACSS2 in tumors, mice xenografted with either high or low ACSS2-expressing isogenic tumors could be given a bolus of ^{13}C-acetate. This would lead to higher fractional acetate labeling in high ACSS2 tumors than in low ACSS2 tumors because, in the latter, tumor cells release more unlabeled acetate. Co-administration of an HDAC inhibitor would reduce the unlabeled acetate in the low ACSS2 xenografts and increase fractional labeling.

In conclusion, our work shows that ACSS2 serves a dual function during oxygen and serum limitation. It facilitates consumption of extracellular acetate as an alternative carbon source, but the increased nuclear localization also enables cells to retain much of their endogenously produced acetate. ACSS2, in essence, “recycles” two-carbon units when nutrient availability is low, which is especially relevant in the poorly perfused, hypoxic areas of the tumor. It allows cells to maintain sufficient acetylation of histones, to prevent initiation of apoptosis, and to maintain growth (Lyssiotis and Cantley, 2014; Lee et al., 2014). This knowledge may inform the ongoing evaluation of ACSS2 as a therapeutic target.

EXPERIMENTAL PROCEDURES

Cell Culture

Cell lines were from the ATCC and routinely passaged in DMEM (HyClone) with 25 mM glucose and 2 mM L-glutamine supplemented with 10% (v/v) fetal bovine serum (FBS)(Gibco) and split at 80% confluence. Cells were routinely checked for mycoplasma. Experiments were performed in DMEM with 10 mM glucose and 2 mM glutamine supplemented with 1% or 10% dialyzed FBS (DFBS) (Sigma). Hypoxia was achieved with a hypoxic chamber (Hypoxystation H35, Whitley Scientific) at 1% O_2, 37°C, and 5% CO_2. Cells and medium were equilibrated in the chamber overnight before the experiment. For experiments, cells were seeded in 6-well plates 24 hr before the experiment to reach 80% confluency at the end of the experiment. 3 ml of medium was used per well. Cell growth was determined by packed cell volume (PCV) (Sartorius Volupac).

Determination of ^{13}C Enrichment in Lipogenic AcCoA

Fatty acid labeling was performed as published previously (Tumanov et al., 2015). Briefly, DMEM with U-^{13}C-glucose (10 mM) and U-^{13}C-glutamine (2 mM) and/or U-^{13}C-acetate (Sigma) was used as indicated. After 48–72 hr of incubation, the medium was aspirated, and cells were washed twice with 2 ml PBS and quenched with 0.75 ml 1:1 v/v PBS:methanol at −20°C. Then, the extraction solvent after cell scraping was transferred to glass tubes, and the total fatty acids (free and lipid-bound) were extracted in 0.5 ml chloroform (−20°C) and dried under nitrogen gas. Lipids were saponified and methylated with 80 μL toluene, 600 μL methanol, and 120 μL methanolic-HCl, followed by vortexing and incubation at 100°C for 60 min. Fatty acid methyl esters were extracted with 400 μL water and 300 μL hexane, and the hexane fraction analyzed by GC-MS (Tumanov et al., 2015). Lipogenic AcCoA ^{13}C enrichment was derived from fatty acid labeling by determining the best fit with computed binomial distributions based on a range of AcCoA labeling enrichments (Kamphorst et al., 2014; Tumanov et al., 2015).

Medium Acetate Quantification

Acetate was derivatized as before (Tumanov et al., 2016). Briefly, 50 μL 1-propanol was added to 200 μL medium in a 2-mL microfuge tube. 40 μL 1 mM $^2H_3$-acetate (Sigma) was added, and the tube was placed on ice in the fume hood, followed by 50 μL pyridine and 5 min of incubation on ice. Next, 100 μL 1 M NaOH was added, followed by 30 μL methyl chloroformate (MCF), vortexing for 20 s, and, finally, addition of 300 μL of tert-butyl methyl ether (MTBE). Samples were vortexed for 2 min and centrifuged at 10,000 × g for 5 min. The top layer was transferred to GC-MS vials and analyzed with an Agilent 7890B GC and an Agilent 7000 Triple Quadrupole GC-MS system with a Phenomenex ZB-1701 column (30 m × 0.25 mm × 0.25 μm). Data were extracted with Agilent Mass Hunter B.06.00 software, from MS intensities of mass-to-charge ratio (m/z) 61 and 63 ions, corresponding to ^{12}C- and ^{13}C-acetate, respectively, which were quantified with $^2H_3$-acetate (m/z of 64).

Metabolite Extraction and Analysis by LC-MS

Cellular metabolites were extracted as reported previously (Mackay et al., 2015). Analysis was performed with a Q-Exactive Orbitrap mass spectrometer (Thermo Scientific) and a Thermo Ultimate 3000 high-performance liquid chromatography (HPLC) system. HPLC consisted of a ZIC-pHILIC column (SeQuant, 150 × 2.1 mm, 5 μm, Merck) with a ZIC-pHILIC guard column (SeQuant, 20 × 2.1 mm) and an initial mobile phase of 20% 20 mM ammonium carbonate (pH 9.4), and 80% acetonitrile. Cell extracts (5 μl) were injected, and metabolites were separated over a 15-min mobile phase gradient, decreasing the acetonitrile to 20%, at a flow rate of 200 μL/min and column temperature of 45°C. Total analysis time was 23 min. The mass range was 75–1,000 m/z at 35,000 resolution (at 200 m/z), with polarity switching for both positive and negative ion analysis. Lock masses were used, and mass accuracy was below 5ppm. Data were processed with MAVEN software (Melamud et al., 2010).

Western Blotting

Protein lysates were prepared in radioimmunoprecipitation assay (RIPA) buffer (Pierce) with protease inhibitor cocktail (Sigma), and total protein concentration was determined by protein assay (Bio-Rad). Proteins were separated using precast NuPAGE gels (Invitrogen, Life Technologies) and transferred onto a nitrocellulose membrane. Protein detection and quantification were done with a LI-COR Odyssey infrared flatbed scanner (LI-COR Biosciences). The primary antibodies used were as follows: anti-ACSS1, 1:1,000 (Sigma, SAB1400745); anti-β-tubulin, 1:5,000 (Sigma, T5201); anti-ACSS2, 1:2,500 (Cell Signaling Technology, 3658S); anti-acetyl-histone H3, 1:1,000 (Millipore, 06-599); anti-acetyl-histone H4, 1:1,000 (Millipore, 06-598); anti-histone H3, 1:5,000 (Active Motif, 39763); and anti-histone H4, 1:5,000 (Active Motif, 61521). Secondary antibodies were IRDye680LT- and IRDye800CW-conjugated (LI-COR Biosciences).

Acid Extraction of Histones and Histone-Bound Acetate

Cells were cultured in 6-well plates for 48 hr under the indicated conditions, followed by washing with cold PBS containing 10 mM sodium butyrate and 50 mM nicotinamide. Nuclei were isolated and histones extracted as described previously (Lee et al., 2014). 15% SDS-PAGE gels were used for separating histones and then transferred onto a nitrocellulose membrane and probed using acetyl-histone-specific antibodies. For GC-MS, isolated histones were hydrolyzed overnight (heating at 95°C overnight in 10 M NaOH) and then neutralized with hydrochloric acid. The resulting free acetate was processed as before.

Immunofluorescence Analysis and Imaging

Cells were maintained for 48 hr on glass coverslips in the indicated medium. For fixation, cells were washed three times with PBS and then incubated for 10 min in 4% paraformaldehyde. Cells were washed three times with PBS and permeabilized for 5 min in PBS containing 0.2% Triton X-100. Following additional washing (three times) in PBS, samples were blocked in PBS with 0.1% Triton X-100 and 5% FBS for 30 min at room temperature (RT). Samples were first incubated with anti-ACSS2 (1:200) antibody (Cell Signaling Technology) and then with anti-rabbit Alexa Fluor 488 secondary antibodies and DAPI, washed, and mounted on a glass coverslip using Dako fluorescence mounting medium. Images were taken on an Olympus FV1000 microscope, and processing was done with ImageJ software. Nuclear-to-cytoplasm staining ratio quantification was done using a macro.

MMTV-PyMT Mice

MMTV-PyMT mice (Guy et al., 1992) were obtained from The Jackson Laboratory. Mammary tumors were measured by caliper twice weekly, and mice were culled humanely when tumors reached the clinical endpoint, according to regulations. Animal experiments were done in accordance with United Kingdom regulations under project license PPL 70/8645, ethical review (University of Glasgow), and EU Directive 2010.

Immunohistochemistry and Image Analysis

Serial sections (4 μm) of paraffin-embedded tumor slices were stained for H&E and immunostained using a Dako autostainer for AceCS1 (ACSS2) (Cell Signaling Technology, 3658S), CD31 (Abcam, ab28364), and CAIX (Novus Biologicals, NB100-417). Slides were analyzed using HALO v2.0 software (Indica Labs). Sections stained for CAIX and ACSS2 were analyzed using Cytonuclear v1.5. Briefly, first hematoxylin staining was set as the nuclear stain and ACSS2 staining as stain 1, and we manually checked for stain separation. ACSS2 staining was specified to localize to the nucleus to get the nuclear staining intensities, and individual cells were assigned to have weak, moderate, or strong nuclear intensities in a given area based on pre-defined threshold intensities. This was done for regions of interest (ROIs) that were manually selected in the CAIX image to represent hypoxic or normoxic areas.

siRNA and Transfections

All siRNAs were from Dharmacon (GE Life Sciences), reconstituted in sterile DNase/RNase-free water to a stock concentration of 20 μM, and transfected using Lipofectamine RNAiMAX reagent (Invitrogen) at a final concentration of 20 nM/well. 48 hr after transfection, cells were seeded for experiments. The following siGENOME siRNAs were used: scrambled RNA control (non-targeting siRNA pool #2, D-001206-14-05), *ACSS2* siRNA #1 (D001206-02), *ACSS2* siRNA #2 (D001206-03), *ACSS1* siRNA #1 (A008549-13), and *ACSS1* siRNA #2 (A008549-14).

Statistical Analyses

Two-tailed Student's t tests and Pearson correlation analysis were done using Graph Pad Prism, version 6. Error bars represent SD of the mean. n is the number of independent wells for each condition and is mentioned in the figure legends for each experiment.

SUPPLEMENTAL INFORMATION

Supplemental Information includes Supplemental Experimental Procedures and six figures and can be found with this article online at http://dx.doi.org/10.1016/j.celrep.2016.12.055.

AUTHOR CONTRIBUTIONS

Conceptualization, V.B. and J.J.K.; Methodology, V.B., S.T., Z.T.S., J.V.V., N.J.V.D.B., G.M., C.N., S.D., K.B., A.V., and E.G.; Investigation, V.B., S.T., E.M., C.N., S.D., and J.J.K.; Writing – Original Draft, V.B. and J.J.K.; Writing – Review & Editing, S.T., E.M., Z.T.S., J.V.V., A.V., and E.G.; Funding Acquisition, E.G. and J.J.K.; Resources, S.T., Z.T.S., J.V.V., S.D., K.B., and A.V.; Supervision, E.G. and J.J.K.

ACKNOWLEDGMENTS

J.J.K is supported by a Cancer Research UK Career Development Fellowship (C50242/A17728). We thank the Cancer Research UK Glasgow Centre (C596/A18076) and the BSU facilities at the Cancer Research UK Beatson Institute (C596/A17196). Eyal Gottlieb is a shareholder and consultant at MetabMed Ltd.

Received: July 29, 2016
Revised: November 16, 2016
Accepted: December 16, 2016
Published: January 17, 2017

REFERENCES

Baeza, J., Smallegan, M.J., and Denu, J.M. (2016). Mechanisms and Dynamics of Protein Acetylation in Mitochondria. Trends Biochem. Sci. *41*, 231–244.

Brown, J.M., and Wilson, W.R. (2004). Exploiting tumour hypoxia in cancer treatment. Nat. Rev. Cancer *4*, 437–447.

Comerford, S.A., Huang, Z., Du, X., Wang, Y., Cai, L., Witkiewicz, A.K., Walters, H., Tantawy, M.N., Fu, A., Manning, H.C., et al. (2014). Acetate dependence of tumors. Cell *159*, 1591–1602.

Currie, E., Schulze, A., Zechner, R., Walther, T.C., and Farese, R.V., Jr. (2013). Cellular fatty acid metabolism and cancer. Cell Metab. *18*, 153–161.

DeBerardinis, R.J., Lum, J.J., Hatzivassiliou, G., and Thompson, C.B. (2008). The biology of cancer: metabolic reprogramming fuels cell growth and proliferation. Cell Metab. *7*, 11–20.

Evertts, A.G., Zee, B.M., Dimaggio, P.A., Gonzales-Cope, M., Coller, H.A., and Garcia, B.A. (2013). Quantitative dynamics of the link between cellular metabolism and histone acetylation. J. Biol. Chem. *288*, 12142–12151.

Falkenberg, K.J., and Johnstone, R.W. (2014). Histone deacetylases and their inhibitors in cancer, neurological diseases and immune disorders. Nat. Rev. Drug Discov. *13*, 673–691.

Fan, J., Kamphorst, J.J., Rabinowitz, J.D., and Shlomi, T. (2013). Fatty acid labeling from glutamine in hypoxia can be explained by isotope exchange without net reductive isocitrate dehydrogenase (IDH) flux. J. Biol. Chem. *288*, 31363–31369.

Fan, J., Krautkramer, K.A., Feldman, J.L., and Denu, J.M. (2015). Metabolic regulation of histone post-translational modifications. ACS Chem. Biol. *10*, 95–108.

Guy, C.T., Cardiff, R.D., and Muller, W.J. (1992). Induction of mammary tumors by expression of polyomavirus middle T oncogene: a transgenic mouse model for metastatic disease. Mol. Cell. Biol. *12*, 954–961.

Kamphorst, J.J., Cross, J.R., Fan, J., de Stanchina, E., Mathew, R., White, E.P., Thompson, C.B., and Rabinowitz, J.D. (2013). Hypoxic and Ras-transformed cells support growth by scavenging unsaturated fatty acids from lysophospholipids. Proc. Natl. Acad. Sci. USA *110*, 8882–8887.

Kamphorst, J.J., Chung, M.K., Fan, J., and Rabinowitz, J.D. (2014). Quantitative analysis of acetyl-CoA production in hypoxic cancer cells reveals substantial contribution from acetate. Cancer Metab. *2*, 23.

Kim, J.W., Tchernyshyov, I., Semenza, G.L., and Dang, C.V. (2006). HIF-1-mediated expression of pyruvate dehydrogenase kinase: a metabolic switch required for cellular adaptation to hypoxia. Cell Metab. *3*, 177–185.

Lee, J.V., Carrer, A., Shah, S., Snyder, N.W., Wei, S., Venneti, S., Worth, A.J., Yuan, Z.F., Lim, H.W., Liu, S., et al. (2014). Akt-dependent metabolic reprogramming regulates tumor cell histone acetylation. Cell Metab. *20*, 306–319.

Lyssiotis, C.A., and Cantley, L.C. (2014). Acetate fuels the cancer engine. Cell *159*, 1492–1494.

Mackay, G.M., Zheng, L., van den Broek, N.J., and Gottlieb, E. (2015). Analysis of Cell Metabolism Using LC-MS and Isotope Tracers. Methods Enzymol. *561*, 171–196.

Mashimo, T., Pichumani, K., Vemireddy, V., Hatanpaa, K.J., Singh, D.K., Sirasanagandla, S., Nannepaga, S., Piccirillo, S.G., Kovacs, Z., Foong, C., et al. (2014). Acetate is a bioenergetic substrate for human glioblastoma and brain metastases. Cell *159*, 1603–1614.

Melamud, E., Vastag, L., and Rabinowitz, J.D. (2010). Metabolomic analysis and visualization engine for LC-MS data. Anal. Chem. *82*, 9818–9826.

Menendez, J.A., and Lupu, R. (2007). Fatty acid synthase and the lipogenic phenotype in cancer pathogenesis. Nat. Rev. Cancer *7*, 763–777.

Metallo, C.M., Gameiro, P.A., Bell, E.L., Mattaini, K.R., Yang, J., Hiller, K., Jewell, C.M., Johnson, Z.R., Irvine, D.J., Guarente, L., et al. (2011). Reductive glutamine metabolism by IDH1 mediates lipogenesis under hypoxia. Nature *481*, 380–384.

Mullen, A.R., Wheaton, W.W., Jin, E.S., Chen, P.H., Sullivan, L.B., Cheng, T., Yang, Y., Linehan, W.M., Chandel, N.S., and DeBerardinis, R.J. (2011). Reductive carboxylation supports growth in tumour cells with defective mitochondria. Nature *481*, 385–388.

Papandreou, I., Cairns, R.A., Fontana, L., Lim, A.L., and Denko, N.C. (2006). HIF-1 mediates adaptation to hypoxia by actively downregulating mitochondrial oxygen consumption. Cell Metab. *3*, 187–197.

Peck, B., Schug, Z.T., Zhang, Q., Dankworth, B., Jones, D.T., Smethurst, E., Patel, R., Mason, S., Jiang, M., Saunders, R., et al. (2016). Inhibition of fatty acid desaturation is detrimental to cancer cell survival in metabolically compromised environments. Cancer Metab. *4*, 6.

Pietrocola, F., Galluzzi, L., Bravo-San Pedro, J.M., Madeo, F., and Kroemer, G. (2015). Acetyl coenzyme A: a central metabolite and second messenger. Cell Metab. *21*, 805–821.

Schug, Z.T., Peck, B., Jones, D.T., Zhang, Q., Grosskurth, S., Alam, I.S., Goodwin, L.M., Smethurst, E., Mason, S., Blyth, K., et al. (2015). Acetyl-CoA synthetase 2 promotes acetate utilization and maintains cancer cell growth under metabolic stress. Cancer Cell *27*, 57–71.

Shi, L., and Tu, B.P. (2015). Acetyl-CoA and the regulation of metabolism: mechanisms and consequences. Curr. Opin. Cell Biol. *33*, 125–131.

Tumanov, S., Bulusu, V., and Kamphorst, J.J. (2015). Analysis of Fatty Acid Metabolism Using Stable Isotope Tracers and Mass Spectrometry. Methods Enzymol. *561*, 197–217.

Tumanov, S., Bulusu, V., Gottlieb, E., and Kamphorst, J.J. (2016). A rapid method for quantifying free and bound acetate based on alkylation and GC-MS analysis. Cancer Metab. *4*, 17.

Wise, D.R., Ward, P.S., Shay, J.E.S., Cross, J.R., Gruber, J.J., Sachdeva, U.M., Platt, J.M., DeMatteo, R.G., Simon, M.C., and Thompson, C.B. (2011). Hypoxia promotes isocitrate dehydrogenase-dependent carboxylation of α-ketoglutarate to citrate to support cell growth and viability. Proc. Natl. Acad. Sci. USA *108*, 19611–19616.

Young, R.M., Ackerman, D., Quinn, Z.L., Mancuso, A., Gruber, M., Liu, L., Giannoukos, D.N., Bobrovnikova-Marjon, E., Diehl, J.A., Keith, B., and Simon, M.C. (2013). Dysregulated mTORC1 renders cells critically dependent on desaturated lipids for survival under tumor-like stress. Genes Dev. *27*, 1115–1131.

Yuan, J., Bennett, B.D., and Rabinowitz, J.D. (2008). Kinetic flux profiling for quantitation of cellular metabolic fluxes. Nat. Protoc. *3*, 1328–1340.

Zhao, S., Torres, A., Henry, R.A., Trefely, S., Wallace, M., Lee, J.V., Carrer, A., Sengupta, A., Campbell, S.L., Kuo, Y.M., et al. (2016). ATP-Citrate Lyase Controls a Glucose-to-Acetate Metabolic Switch. Cell Rep. *17*, 1037–1052.

Cell Reports, Volume *18*

Supplemental Information

Acetate Recapturing by Nuclear Acetyl-CoA Synthetase 2 Prevents Loss of Histone Acetylation during Oxygen and Serum Limitation

Vinay Bulusu, Sergey Tumanov, Evdokia Michalopoulou, Niels J. van den Broek, Gillian MacKay, Colin Nixon, Sandeep Dhayade, Zachary T. Schug, Johan Vande Voorde, Karen Blyth, Eyal Gottlieb, Alexei Vazquez, and Jurre J. Kamphorst

Experimental procedures

Estimation of acetate exchange fluxes

We denote by C, C_0, and C_2 the concentration of total acetate, ^{12}C acetate and U-^{13}C-acetate in the culture medium, respectively. We denote by c_0 the intracellular fraction of ^{12}C acetate and by V the packed cell volume. We denote by u, r, e=r-u the flux per cell volume of acetate uptake, release and exchange, respectively. We model the time dependent changes in the extracellular acetate concentration with the first order differential equations

(1) $$\frac{dC}{dt} = rV - uV$$

(2) $$\frac{dC_0}{dt} = rVc_0 - uV\frac{C_0}{C}$$

Integrating these equations over time, from the time that [$^{13}C_2$]-Acetate was added to the culture medium (t=0) to the final time (T) we obtain

(3) $$e = r - u$$

(4) $$xe = ar - bu$$

where

(5) $$e = \frac{C(T) - C(0)}{A}$$

(6) $$x = \frac{C_0(T) - C_0(0)}{C(T) - C(0)}$$

(7) $$A = \int_0^T dtV(t)$$

(8) $$a = \frac{1}{A}\int_0^T dtV(t)c_0(t)$$

(9) $$b = \frac{1}{A}\int_0^T dtV(t)C_0(t)/C(t)$$

Equation (5) is the definition of exchange rate. x in Eq. 6 quantifies the change in extracellular ^{12}C acetate relative to the change in total acetate. A (Eq. 7) is the area under the packed cell volume curve. a and b (Eqs. 8 and 9) are the average

intracellular and extracellular ^{12}C fractions, weighted by packed cell volume. Since the only source of ^{13}C acetate is the medium, the ^{13}C intracellular acetate fraction can be at most as large as the extracellular ^{13}C fraction and, therefore, the ^{12}C intracellular acetate fraction must be larger than the extracellular ^{12}C fraction:

(10) $$a > b$$

Solving the linear system of equations (3-4) for u and r we obtain

(11) $$u = e\frac{x-a}{a-b}$$

(12) $$r = e\frac{x-b}{a-b}$$

Since u and r are by definition nonnegative (larger or equal to zero), from equations 10-12, and the fact that a by definition must be smaller than 1, it follows that

(13) $$b < a < \min(1, x)$$

Parameter estimation

The parameters A, e, x and b can be estimated from experimental measurements of V, C and C_0 at different time points. The integrals (7) and (9) were estimated using the trapezium approximation. Given the experimental errors and lack of an experimental estimate of a, we sampled parameters using the following approach. We took V as measured and sampled C and C_0 from a Normal distribution with mean and standard deviation as measured. a was sampled uniformly from the range indicated by equation 13. Parameter sets (V,C,C_0,a) that did not satisfy (13) were rejected. Statistics was made over 1,000 sets of (V,C,C_0,a).

Estimation of fatty acid synthesis flux

The palmitate mass isotope distribution (MID) is modelled as follows. Palmitate MIDs are first corrected for natural abundance of ^{13}C. There is a M+0 pool of palmitate that was present in the cells at the time the tracer (or tracers) was added to the culture medium. The fraction of this pool at a given time t is denoted by ε_t. There is a pool of palmitate coming from *de novo* synthesis. This pool has a binomial MID distribution on n=8 acetyl groups each labelled at both carbons with probability p. The fraction of this pool at a given time t is denoted by $(1-\varepsilon_t)y$. Finally, there is a M+0 pool of palmitate coming from other sources. The fraction of this pool at a given time

t is denoted by $(1-\varepsilon_t)(1-y)$. Putting all together the palmitate MID distribution is given by

(Eq 1) $$x_i = \begin{cases} \varepsilon + (1-\varepsilon)\left[y(1-p)^n + 1 - y\right] & i = 0 \\ (1-\varepsilon)y\begin{pmatrix} n \\ m \end{pmatrix} p^m (1-p)^{n-m} & i = 2m \\ 0 & i = 2m+1 \end{cases}$$

The residual pool ε_t decays exponentially in time [X]

(Eq 2) $$\varepsilon_t = e^{-(k+\mu)t}$$

Where k is the palmitate turnover rate per unit of palmitate and μ is the cell population proliferation rate. From (Eq 1) and (Eq 2) we obtain

(Eq 3) $$x_i = \begin{cases} \alpha + (1-\alpha)(1-p)^n & i = 0 \\ (1-\alpha)\begin{pmatrix} n \\ m \end{pmatrix} p^m (1-p)^{n-m} & i = 2m \\ 0 & i = 2m+1 \end{cases}$$

where

(Eq 4) $$\alpha = 1 - (1 - e^{-(k+\mu)t})y$$

Given the palmitate MID (x_i) at a given time point, we calculate the mean squared error estimate of α and p. Given two time point estimates of α, we calculate the mean squared error estimate of $k+\mu$ and y. We fix one time point to an early time point where we already observe a saturation of p to a steady state value. The second time point run across measurements at later time points, obtaining time point estimates of $k+\mu$ and y. If these time points estimates do not change significantly in time we can warranty that with respect to the palmitate balance cells are in an approximate steady state. Finally, having estimated $k+\mu$, we estimate the palmitate synthesis rate as [X]

(Eq 5) $$f = C(k+\mu)$$

where C is the concentration of palmitate in cells.

ACSS1 overexpression

Human ACSS1 ORF sequence was obtained from NCBI database and was codon optimized and custom synthesized using GeneArt® Gene Synthesis (Life technologies). The sequence was cloned into pLIX mammalian expression vector obtained from Addgene (Addgene (https://www.addgene.org/41393/) and verified by DNA sequencing. Lentiviruses were produced by co-transfecting HEK293T cells with pLIX or pLIX+ACSS1 and the packaging plasmids. Supernatants containing virus were collected 24 and 48 hr after transfection, mixed with polybrene and used to infect MDA-MB-468 cells. Stable clones of MDA-MB-468 cells were selected for puromycin resistance and expression of ACSS1 was induced and maintained by the addition of 5μg ml^{-1} of doxycycline.

Total RNA extraction and quantitative RT-PCR

Total RNA was extracted using RNeasy kit (Qiagen) according to manufacturer's instructions. First strand cDNA was synthesized using 1 μg of total RNA using QuantiTect Reverse Transcription kit (Qiagen) according to manufacturer's instructions. Quantitative PCR (qPCR) was performed using SYBR® Green PCR Master Mix (BioRad) and primers for ACSS1 or for actin on a BioRad CFX96 real time PCR detection system (BioRad). Relative mRNA expression was calculated using the comparative Ct method after normalization to actin control.

SUPPLEMENTARY FIGURES

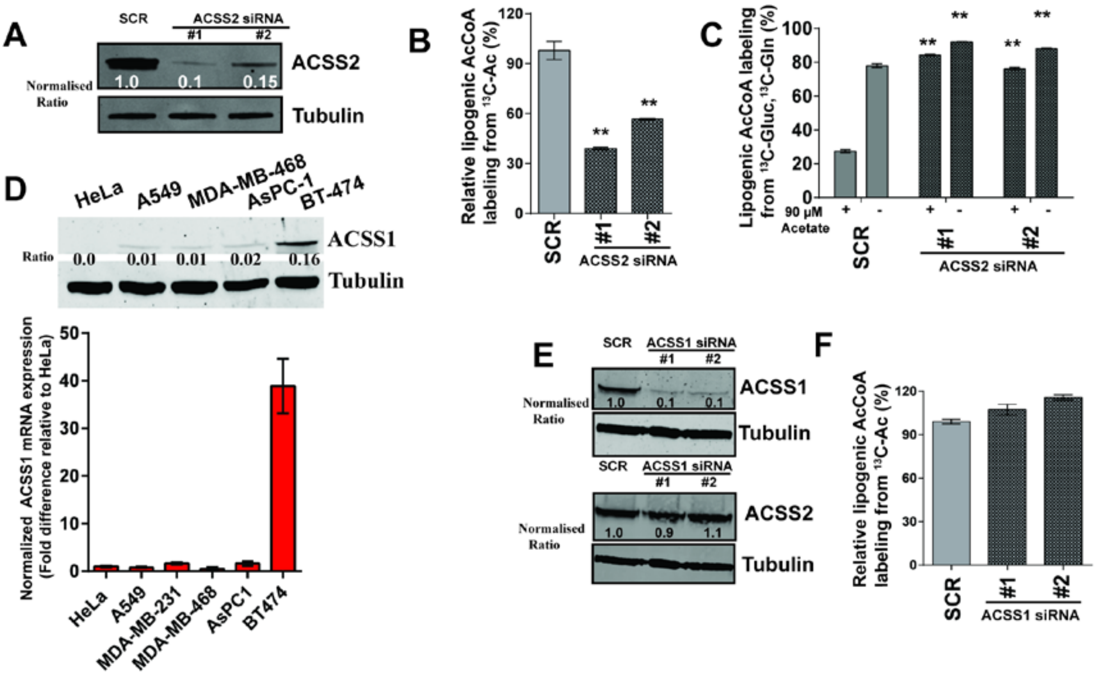

Figure S1. Related to Figure 1 (A) Western blot of ACSS2 in BT-474 cells transfected with either the scrambled RNA (SCR) or 2 independent ACSS2 siRNAs (#1 and #2). Expression levels of ACSS2 were normalized to tubulin and expressed relative to SCR control. (B) Percent ^{13}C labeling of lipogenic AcCoA from 90 µM U-^{13}C-acetate (Ac) in hypoxic BT-474 cells, transfected with either SCR or ACSS2 siRNAs (48 h labeling). (C) Percent ^{13}C labeling of lipogenic AcCoA from U-^{13}C-glucose and U-^{13}C-glutamine in hypoxic MDA-MB-468 cells with or without 90µM ^{12}C-acetate supplemented to the medium (48 h incubation). (D) Western blot analysis of ACSS1 expression in various cell lines, cultured under hypoxia (1% O_2) and low (1%) dialyzed serum for 48h. ACSS1 expression in each lane was normalized to tubulin and expressed as a ratio. Quantitative RT-PCR analysis of ACSS1 expression. Data was normalized to actin control and expressed relative to HeLa cells. (E) Western blot analysis of ACSS1 and ACSS2 expression in BT-474 cells, transfected with either the scrambled RNA (SCR) or 2 independent ACSS1 siRNAs (#1 and #2). Expression levels of ACSS1 or ACSS2 were normalized to tubulin in each lane and expressed as relative to SCR control. (F) Percent ^{13}C labeling of lipogenic AcCoA from 500µM U-^{13}C-acetate (Ac) in hypoxic BT-474 cells transfected with SCR or independent ACSS1 siRNAs (48 h labeling). For B, C, D and F, data are means ± SD (n=3). ** $p < 0.01$.

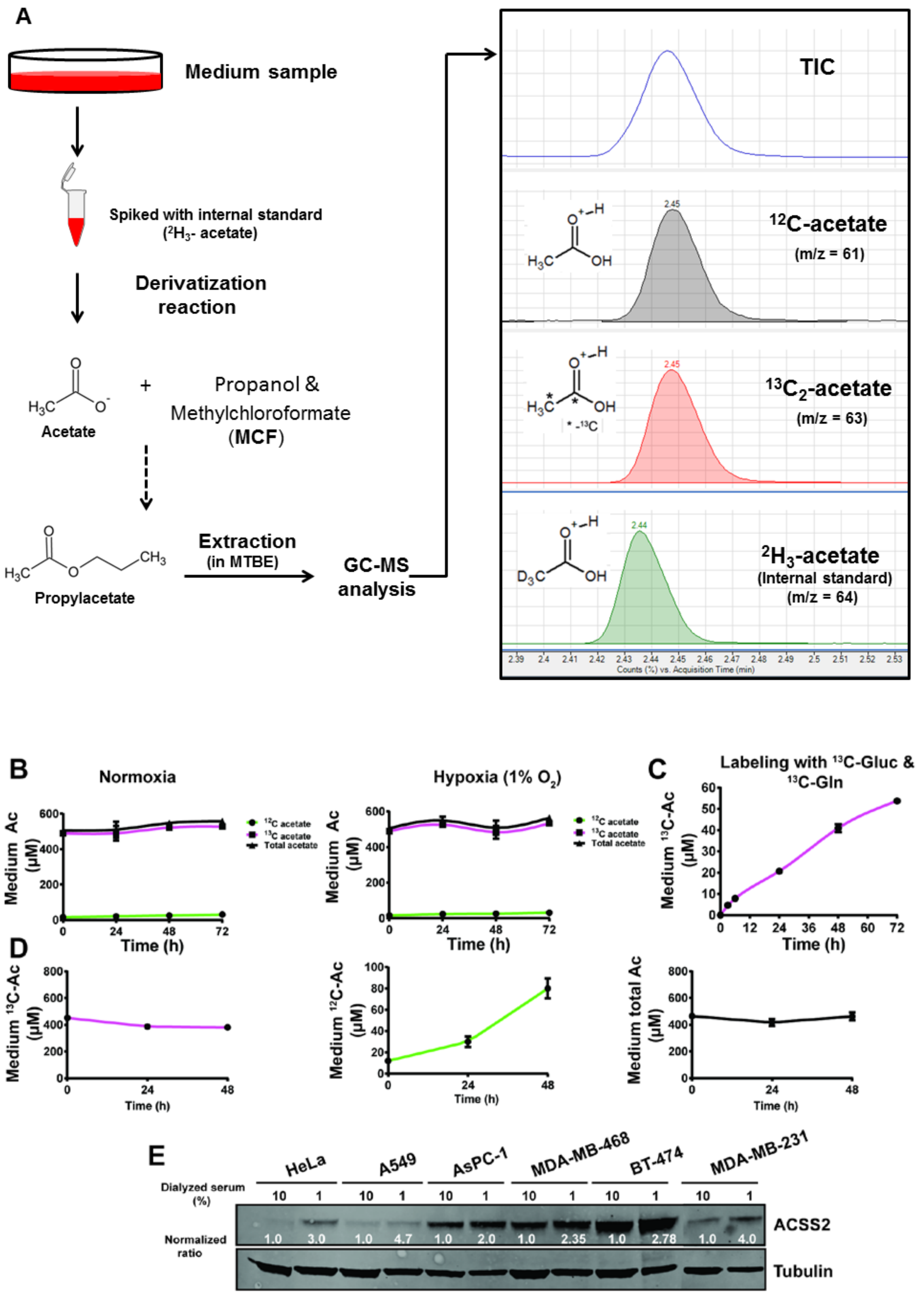

Figure S2. Related to Figure 2. (A) Scheme of acetate quantification from media samples by GC/MS. Culture medium was sampled and spiked with $^{2}H_{3}$-acetate as an internal standard and then derivatized to propyl-acetate in a methyl chloroformate

(MCF)-driven reaction (details in methods). Following extraction in MTBE (Ter-butyl methyl ether), samples were analyzed by GC/MS. Shown are the total ion current (TIC) and the integrated extracted ion chromatograms for ^{12}C-acetate (m/z 61), $^{13}C_2$-acetate (m/z 63) and $^{2}H_3$-acetate (m/z 64), which are generated from propyl-acetate by the electron impact ionization. Numbers on top of the peaks represent retention times. (B) Time course of U-^{13}C-acetate, ^{12}C-acetate and total acetate concentrations in experiment medium (10% dialyzed serum) incubated without cells. (C) Time course of ^{13}C acetate concentration in the medium of MDA-MB-468 cells incubated with U-^{13}C-glucose and U-^{13}C-glutamine. (D) Time course of U-^{13}C-acetate, ^{12}C-acetate and total acetate concentrations for hypoxic MDA-MB-468 cells cultured in medium containing physiological glucose (5.5 mM) and glutamine (0.65 mM), and additionally 10% dialyzed serum and 500 µM U-^{13}C-Acetate. (E) ACSS2 expression in panel of cancer cell lines, under hypoxia (48 h incubation) and high (10%) or low (1%) dialyzed serum. Band intensities of ACSS2 in each lane were first normalized to tubulin and then expressed as ratio normalized to 10% dialyzed serum condition for each cell line to see the fold increase in 1% dialyzed serum condition. For B-D, data are means ± SD (n=3).

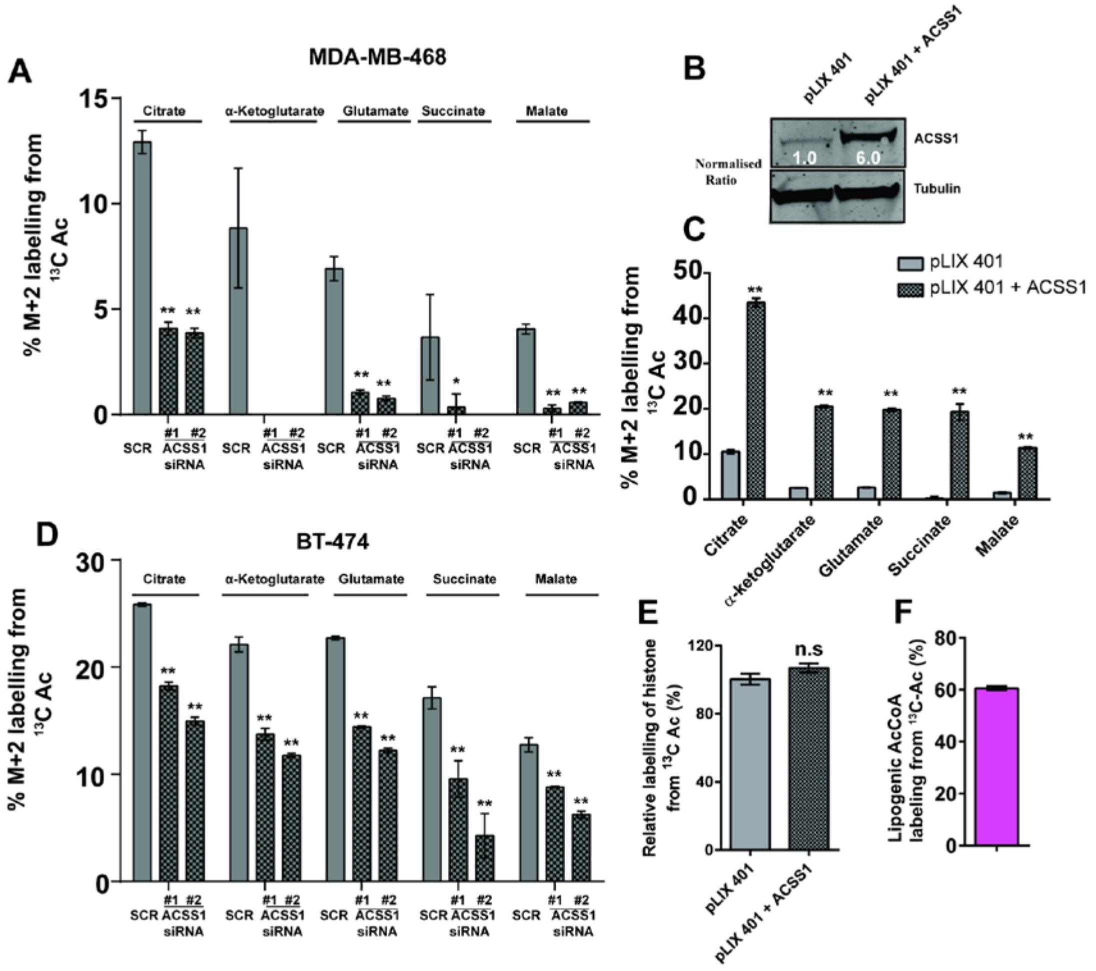

Figure S3. Related to Figure 3. ACSS1 knockdown by two independent siRNAs (#1,#2) decreases labelling of TCA cycle intermediates in MDA-MB-468 (A) and BT-474 (D) cells from 500μM U-^{13}C-acetate. (B) Western blot of ACSS1 in MDA-MB-468 cells stably transfected with either empty vector (pLIX 401) or vector with ACSS1 ORF (pLIX 401 + ACSS1). Cells were maintained in 5μg ml^{-1} doxycycline for induction of ACSS1 expression. (C) Labelling of TCA cycle intermediates for 48h from 500μM U-^{13}C-acetate in MDA-MB-468 cells stably transfected with pLIX 401 or pLIX 401+ ACSS1. Cells were maintained in 5μg ml^{-1} doxycycline for induction of ACSS1 expression. (E) Relative labelling of histone bound acetate from 500μM U-^{13}C-acetate in MDA-MB-468 cells stably transfected with pLIX 401 or pLIX 401+ ACSS1. Cells were maintained in 5μg ml^{-1} doxycycline for induction of ACSS1 expression. (F) Percent ^{13}C labeling of lipogenic AcCoA from 500μM U-^{13}C-acetate (Ac) in MDA-MB-468 cells grown under hypoxia and low serum conditions (48 h labeling). All data are means ± SD (n=3), * $p < 0.05$, ** $p < 0.01$.

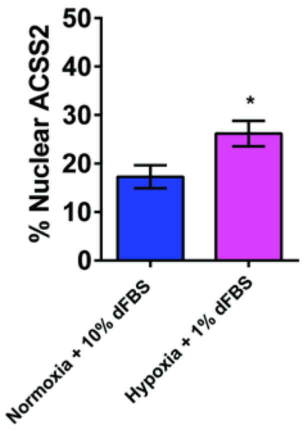

Figure S4. Related to Figure 4. Quantification of the nuclear fraction of ACSS2 (%) in BT-474 cells cultured under normoxia and 10% serum or hypoxia and 1% serum conditions for 48h. Data are means and SD of 3 independent experiments (8 images per experiment). * $p < 0.05$.

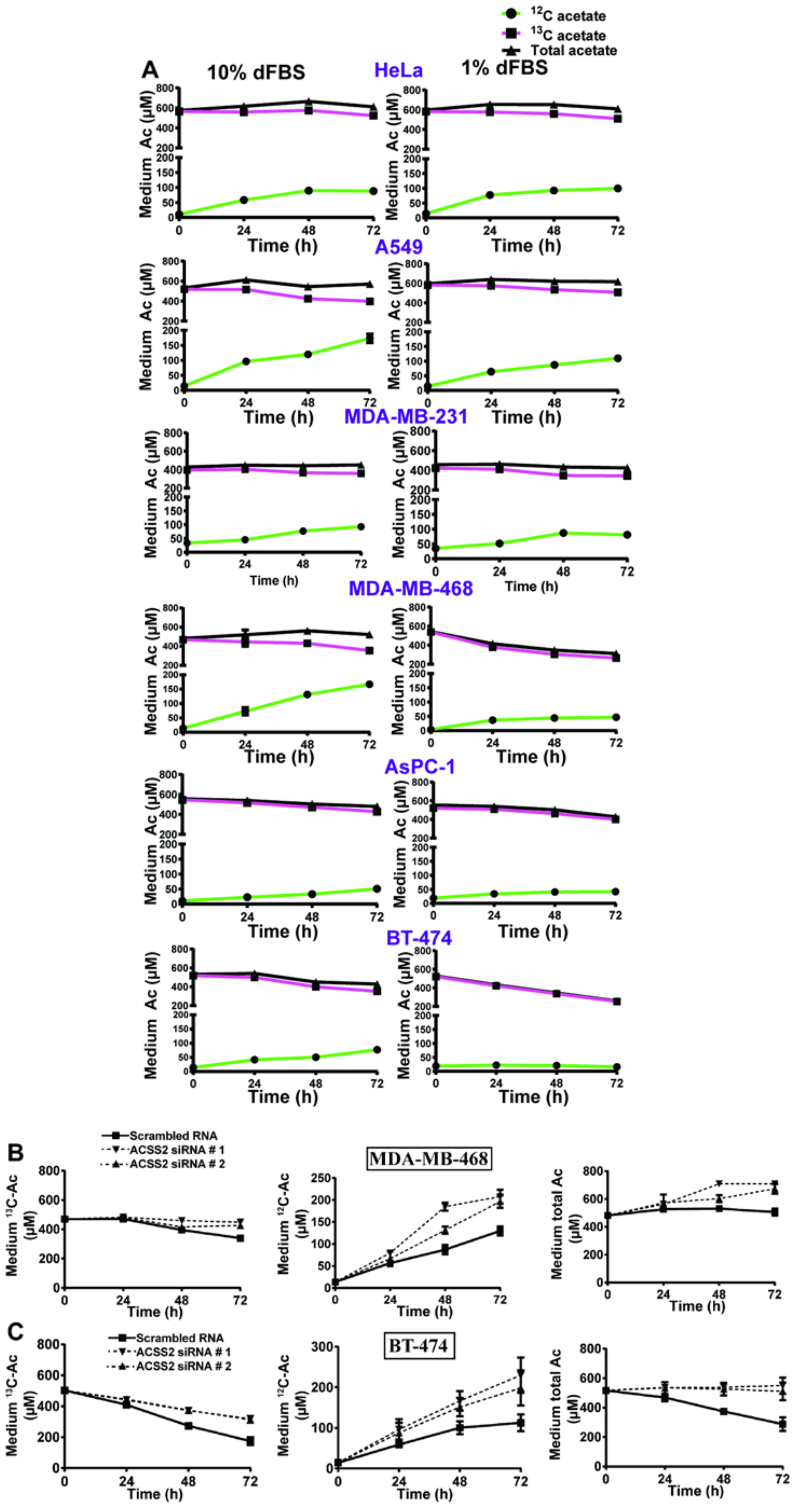

12C acetate
13C acetate
Total acetate
A
10% dFBS
HeLa
1% dFBS
A549
MDA-MB-231
MDA-MB-468
AsPC-1
BT-474
Medium Ac (μM)
Time (h)
B
Scrambled RNA
ACSS2 siRNA # 1
ACSS2 siRNA # 2
MDA-MB-468
Medium 13C-Ac (μM)
Medium 12C-Ac (μM)
Medium total Ac (μM)
C
BT-474

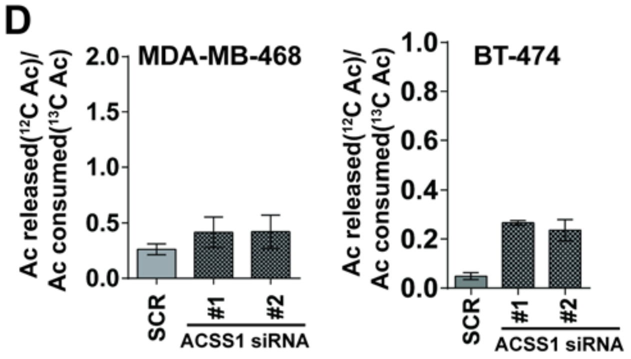

Figure S5. Related to Figure 5. (A) Time course of U-^{13}C-acetate, ^{12}C-acetate and total acetate concentrations for panel of cancer cell lines incubated under hypoxic conditions in medium supplemented with 10% or 1% dialyzed serum. (B) Acetate exchange by MDA-MB-468 cells following SCR or ACSS2 siRNAs under hypoxic conditions in medium supplemented with 10% dialyzed serum and 500μM U-^{13}C-acetate. (C) Same but for BT-474 cells. (D) Acetate exchange by MDA-MB-468 and BT-474 cells following SCR or ACSS1 siRNAs under hypoxic conditions in medium supplemented with 1% dialyzed serum and 500μM U-^{13}C-acetate. All data are means ± SD (n=3).

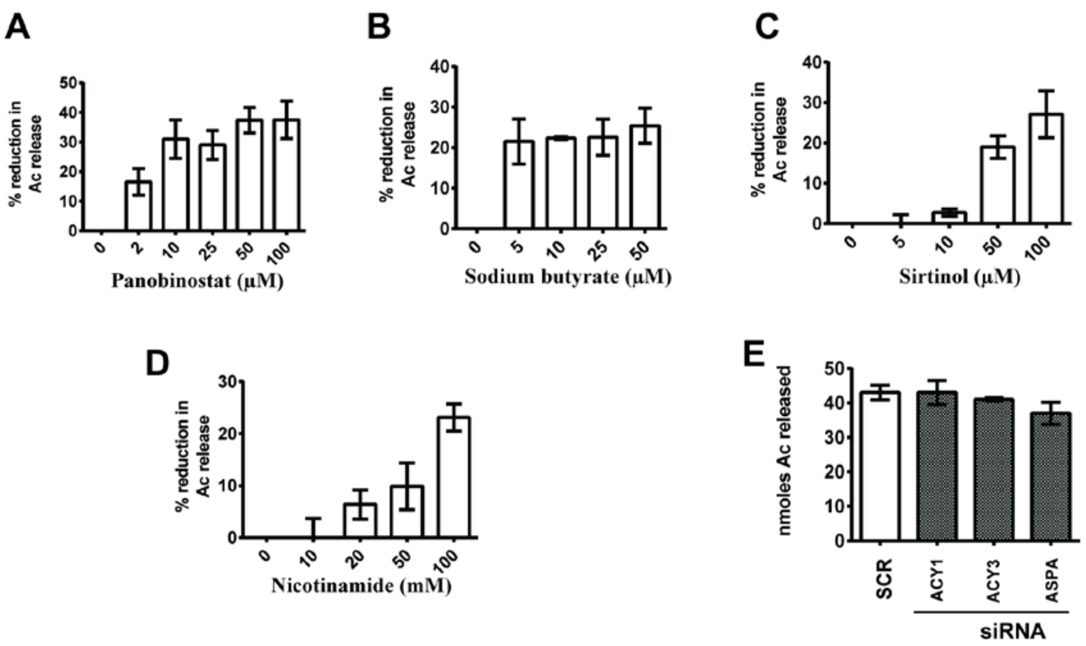

Figure S6. Related to Figure 6. (A) Percent reduction in acetate released by MDA-MB-468 cells treated with increasing concentrations of panobinostat. Cells were incubated with indicated concentrations of panobinostat for 6h in hypoxia in medium containing 1% dialyzed serum and 500µM U-^{13}C-acetate. Data is expressed as % reduction in ^{12}C acetate release relative to controls. (B) Same as (A), but with sodium butyrate treatment at indicated concentration (C) Same as (A), but with sirtinol treatment at indicated concentrations. (D) Same as (A), but with nicotinamide treatment at indicated concentrations. (E) Plot of percent reduction in acetate released by MDA-MB-468 cells treated with different siRNAs of amino acid deacetylases. Cells were transfected with either the scrambled siRNA or different SMARTpool siRNAs targeting aminoacylase 1 (ACY1), aminoacylase 3 (ACY3) or aspartoacylase (ASPA). Cells were incubated for 6h in experiment DMEM with 1% dialyzed serum. For A-E, data are means ± SD (n=3).
